# Clinical trial of ABCB5^+^ mesenchymal stem cells for recessive dystrophic epidermolysis bullosa

**DOI:** 10.1172/jci.insight.151922

**Published:** 2021-11-22

**Authors:** Dimitra Kiritsi, Kathrin Dieter, Elke Niebergall-Roth, Silvia Fluhr, Cristina Daniele, Jasmina Esterlechner, Samar Sadeghi, Seda Ballikaya, Leoni Erdinger, Franziska Schauer, Stella Gewert, Martin Laimer, Johann W. Bauer, Alain Hovnanian, Giovanna Zambruno, May El Hachem, Emmanuelle Bourrat, Maria Papanikolaou, Gabriela Petrof, Sophie Kitzmüller, Christen L. Ebens, Markus H. Frank, Natasha Y. Frank, Christoph Ganss, Anna E. Martinez, John A. McGrath, Jakub Tolar, Mark A. Kluth

**Affiliations:** 1 Department of Dermatology, Medical Center – University of Freiburg, Faculty of Medicine, Freiburg, Germany.; 2 RHEACELL GmbH & Co. KG, Heidelberg, Germany.; 3 TICEBA GmbH, Heidelberg, Germany.; 4 EB House Austria, Department of Dermatology and Allergology, University Hospital of the Paracelsus Medical University Salzburg, Salzburg, Austria.; 5 Department of Genetics at Necker Hospital and; 6 Department of Dermatology at Saint-Louis Hospital, INSERM UMR; 1163, Laboratory of Genetic Skin Diseases, IMAGINE Institute for Genetic Diseases, University of Paris, Paris, France.; 7 Genodermatosis Unit and; 8 Dermatology Unit and Genodermatosis Unit, Genetics and Rare Diseases Research Division, Bambino Gesù Children’s Hospital, IRCSS, Rome, Italy.; 9 Department of Dermatology, Reference Center for Rare Skin Diseases MAGEC, St. Louis Hospital, Paris, France.; 10 St. John’s Institute of Dermatology, Guy’s Hospital, King’s College London, London, United Kingdom.; 11 Department of Dermatology, Great Ormond Street Hospital NHS Foundation Trust, London, United Kingdom.; 12 Division of Blood and Marrow Transplantation and Cellular Therapy, Department of Pediatrics, University of Minnesota M Health Fairview Masonic Children’s Hospital, Minneapolis, Minnesota, USA.; 13 Department of Dermatology, Brigham and Women’s Hospital, Harvard Medical School, Boston, Massachusetts, USA.; 14 Transplant Research Program, Boston Children’s Hospital, Harvard Medical School, Boston, Massachusetts, USA.; 15 Harvard Stem Cell Institute, Harvard University, Cambridge, Massachusetts, USA.; 16 School of Medical and Health Sciences, Edith Cowan University, Perth, Western Australia, Australia.; 17 Department of Medicine, VA Boston Healthcare System, Boston, Massachusetts, USA.; 18 Division of Genetics, Brigham and Women’s Hospital, Harvard Medical School, Boston, Massachusetts, USA.

**Keywords:** Clinical Trials, Stem cells, Adult stem cells

## Abstract

**BACKGROUND:**

Recessive dystrophic epidermolysis bullosa (RDEB) is a rare, devastating, and life-threatening inherited skin fragility disorder that comes about due to a lack of functional type VII collagen, for which no effective therapy exists. ABCB5^+^ dermal mesenchymal stem cells (ABCB5^+^ MSCs) possess immunomodulatory, inflammation-dampening, and tissue-healing capacities. In a *Col7a1*^–/–^ mouse model of RDEB, treatment with ABCB5^+^ MSCs markedly extended the animals’ lifespans.

**METHODS:**

In this international, multicentric, single-arm, phase I/IIa clinical trial, 16 patients (aged 4–36 years) enrolled into 4 age cohorts received 3 i.v. infusions of 2 × 10^6^ ABCB5^+^ MSCs/kg on days 0, 17, and 35. Patients were followed up for 12 weeks regarding efficacy and 12 months regarding safety.

**RESULTS:**

At 12 weeks, statistically significant median (IQR) reductions in the Epidermolysis Bullosa Disease Activity and Scarring Index activity (EBDASI activity) score of 13.0% (2.9%–30%; *P* = 0.049) and the Instrument for Scoring Clinical Outcome of Research for Epidermolysis Bullosa clinician (iscorEB‑c) score of 18.2% (1.9%–39.8%; *P* = 0.037) were observed. Reductions in itch and pain numerical rating scale scores were greatest on day 35, amounting to 37.5% (0.0%–42.9%; *P* = 0.033) and 25.0% (–8.4% to 46.4%; *P* = 0.168), respectively. Three adverse events were considered related to the cell product: 1 mild lymphadenopathy and 2 hypersensitivity reactions. The latter 2 were serious but resolved without sequelae shortly after withdrawal of treatment.

**CONCLUSION:**

This trial demonstrates good tolerability, manageable safety, and potential efficacy of i.v. ABCB5^+^ MSCs as a readily available disease-modifying therapy for RDEB and provides a rationale for further clinical evaluation.

**TRIAL REGISTRATION:**

Clinicaltrials.gov NCT03529877; EudraCT 2018-001009-98.

**FUNDING:**

The trial was sponsored by RHEACELL GmbH & Co. KG. Contributions by NYF and MHF to this work were supported by the NIH/National Eye Institute (NEI) grants RO1EY025794 and R24EY028767.

## Introduction

Recessive dystrophic epidermolysis bullosa (RDEB) is a rare, devastating, and life-threatening inherited skin fragility disorder. RDEB is caused by biallelic mutations in the *Col7a1* gene coding for type VII collagen, the main component of the anchoring fibrils that ensure adherence of the epidermis to the dermis within the basement membrane zone (1, 2). Lack of functional type VII collagen is associated with extremely weakened cutaneous mechanical stability, which manifests with blistering, chronic and recurrent wounds, erosions, and excessive scarring of the skin, accompanied by significant pruritus and pain and an exceptionally high risk for developing aggressive forms of squamous cell carcinoma (3–5). In addition, patients suffer from various extracutaneous manifestations, most frequently from esophageal and other gastrointestinal mucosal scarring and corneal erosions (6). Available treatment options are limited to extensive wound management, infection control, prevention of skin trauma, and palliative treatment of complications (3, 7), leaving the patients with a highly impaired quality of life (8) and a strongly increased mortality risk from skin cancer (5).

Over the past 2 decades, several treatment strategies for RDEB have been suggested and investigated (9–12), yet cure or even effective symptom relief have not been achieved. Much research focuses on strategies targeting the genetic defect at the protein, mRNA, or DNA level (12–15). However, while gene correction–based therapy approaches have shown promising, albeit variable, first results in small early-phase clinical trials (16–18), such therapies are associated with complex challenges relating to technological issues, oncogenic potential, immune reactions, and maintenance of the therapeutic effect (12, 14, 15). Thus, it is unclear how quickly curative EB treatments can be implemented in clinical routine care (12, 19, 20).

The unmet desperate need for symptom relief in RDEB has focused research on disease-modifying strategies (21). Such approaches build upon the accumulating evidence that the systemic impact of inflammatory signal cascades associated with the persistent, intrinsic proinflammatory state of RDEB skin (22, 23) significantly contributes to disease severity and complications (24–26). Considering RDEB as a systemic inflammatory disease rather than a skin-limited disorder (25, 26) has propelled new investigations. Among several signaling pathways involved in the inflammatory pathogenesis of RDEB, a substantial contribution was ascribed to persistent mechanical and/or oxidative stress–induced release of IL‑1β by epidermal keratinocytes, which is observed in severe skin inflammatory diseases including RDEB (24). Beyond its local effects on surrounding cells that contribute to sustained skin inflammation, excessively released IL‑1β can spill over in the systemic circulation of patients with RDEB, affect remote organs, and contribute to life-threatening RDEB complications such as amyloidosis and kidney and heart involvement (24–27).

Among other immune-modulating strategies to treat RDEB, including small-molecule agents (11) and hematopoietic progenitor cell transplantation (28), systemic administration of allogeneic mesenchymal stem cells (MSCs) has emerged as a potential, comparatively well-tolerated treatment option (29–33). Transplanted MSCs migrate to injured tissue sites (such as RDEB skin; refs. 34, 35), where they can adaptively respond to biological signals associated with inflammation and injury (36). A hallmark feature of MSCs is their capacity to dampen IL‑1β–driven inflammation by adaptive release of IL‑1 receptor antagonist (IL‑1RA) (37). Recently, a skin-resident immunomodulatory MSC population (38–40), marked by the ATP-Binding Cassette Transporter, Subfamily B, Member 5 (ABCB5; ABCB5^+^ dermal MSCs, ABCB5^+^ MSCs; ref. 41), has been shown to promote healing of chronic wounds after therapeutic administration in preclinical and clinical studies (40, 42, 43). The observed effects could be attributed to IL‑1RA released by the MSCs, which shifted the prevalence of proinflammatory M1 macrophages toward antiinflammatory, repair-promoting M2 macrophages in the wound tissue (40). Moreover, in a *Col7a1*^–/–^ mouse model of RDEB, systemic administration of ABCB5^+^ MSCs reduced RDEB pathology and markedly prolonged the animals’ lifespans via significant reduction of skin infiltration of proinflammatory M1 macrophages (44).

**Figure 1:**
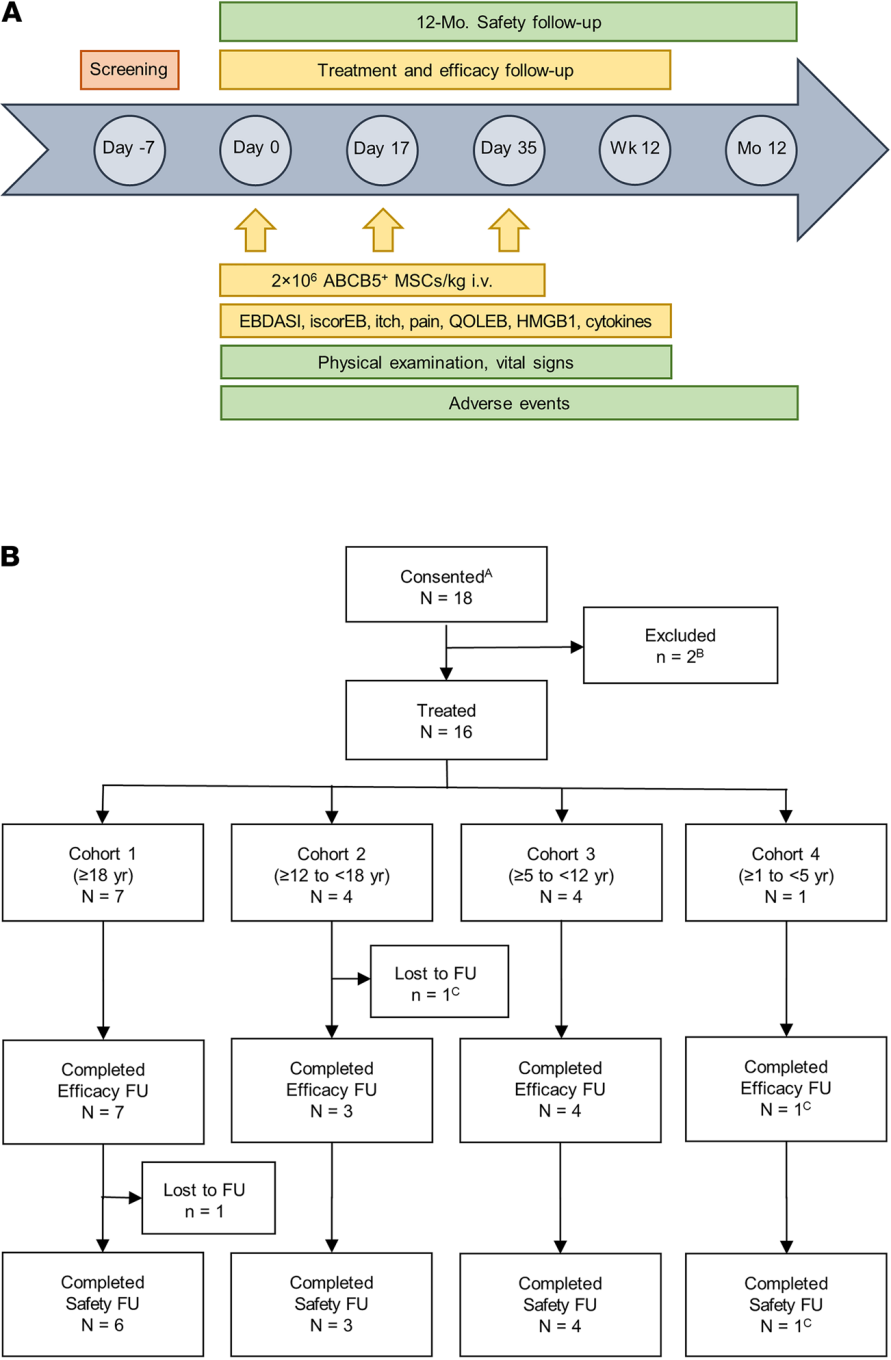
Study summary. (**A** and **B**) Trial design and trial flow chart. ^A^Signed the informed consent form. ^B^Failed to attend the screening visit (due to poor general health, *n* = 1) or day 0 visit (due to travel restrictions associated with the COVID‑19 pandemic, *n* = 1). ^C^Patient was prematurely withdrawn from treatment due to occurrence of a hypersensitivity reaction during the second cell infusion. FU, follow-up.

ABCB5^+^ MSCs can be supplied as an off-the-shelf available, advanced-therapy medicinal product (ATMP) that contains a highly pure cell population with confirmed, standardized antiinflammatory (IL‑1RA secretion) potency (45) and has shown an uncritical safety profile after i.v. single- and repeated-dose application in preclinical studies (46). Here, we report on a phase I/IIa clinical trial of skin-derived allogeneic ABCB5^+^ MSCs to evaluate their safety and potential efficacy in patients with RDEB (Figure 1A).

## Results

### Patients.

**Figure 2:**
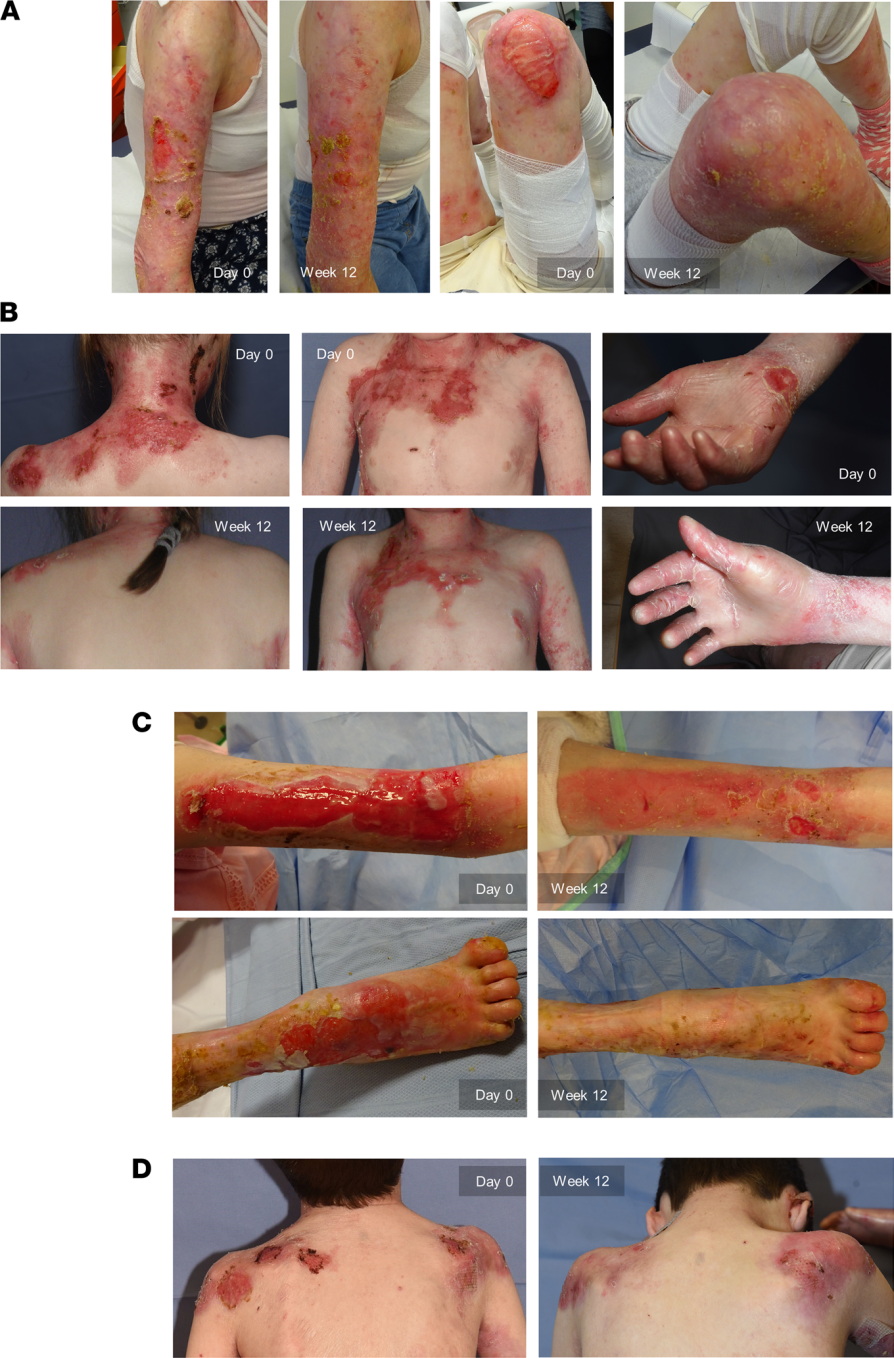
Representative photographs of patients at baseline (Day 0) and after 3 infusions of ABCB5^+^ MSCs (Week 12). (**A**) Right lateral upper arm and right knee of a 24‑year-old Female patient (cohort 1). (**B**) Shoulder/neck area (back and front) and right hand of a 13‑year-old Female patient (cohort 2). (**C**) Right lateral upper arm and dorsum of the right foot of a 13‑year-old Female patient (cohort 2). (**D**) Back shoulder area of a 9‑year-old Male patient (cohort 3). All patients had consented to publication of their photographs.

Between February 2019 and March 2020, 18 patients consented to participate in the trial (Figure 1B). Two patients were excluded because they failed to attend the screening or day 0 visit. The remaining 16 patients (7 Male, 9 Female) were found eligible and were assigned in a staggered fashion to 4 age cohorts as follows: 7 in cohort 1 (≥ 18 years), 4 in cohort 2 (≥ 12 to < 18 years), 4 in cohort 3 (≥ 5 to < 12 years), and 1 in cohort 4 (≥ 1 to < 5 years) (Figure 1B). For baseline characteristics, see Table 1. For all patients, previous or ongoing multiorgan RDEB involvement was reported, most frequently affecting the skin (16 patients), gastrointestinal tract (15 patients), and hematological/lymphatic system (14 patients). The most frequent concurrent diagnosis was iron deficiency, affecting 11 patients, 7 of whom had developed anemia. Representative photographs of patients at baseline and at the end of the 12‑week treatment and efficacy follow-up period are shown in Figure 2.

### Protocol adherence.

In 2 of the 16 (12.5%) treated patients, treatment was prematurely terminated due to occurrence of a hypersensitivity reaction during the second infusion. Both patients received an incomplete second dose and no third dose (Figure 1B). Accordingly, as defined in the trial protocol, efficacy assessments are reported on both the full analysis set (FAS, *n* = 16) and the per-protocol set (PP), from which these 2 patients were excluded (*n* = 14).

### Changes in EBDASI scores.

**Figure 3:**
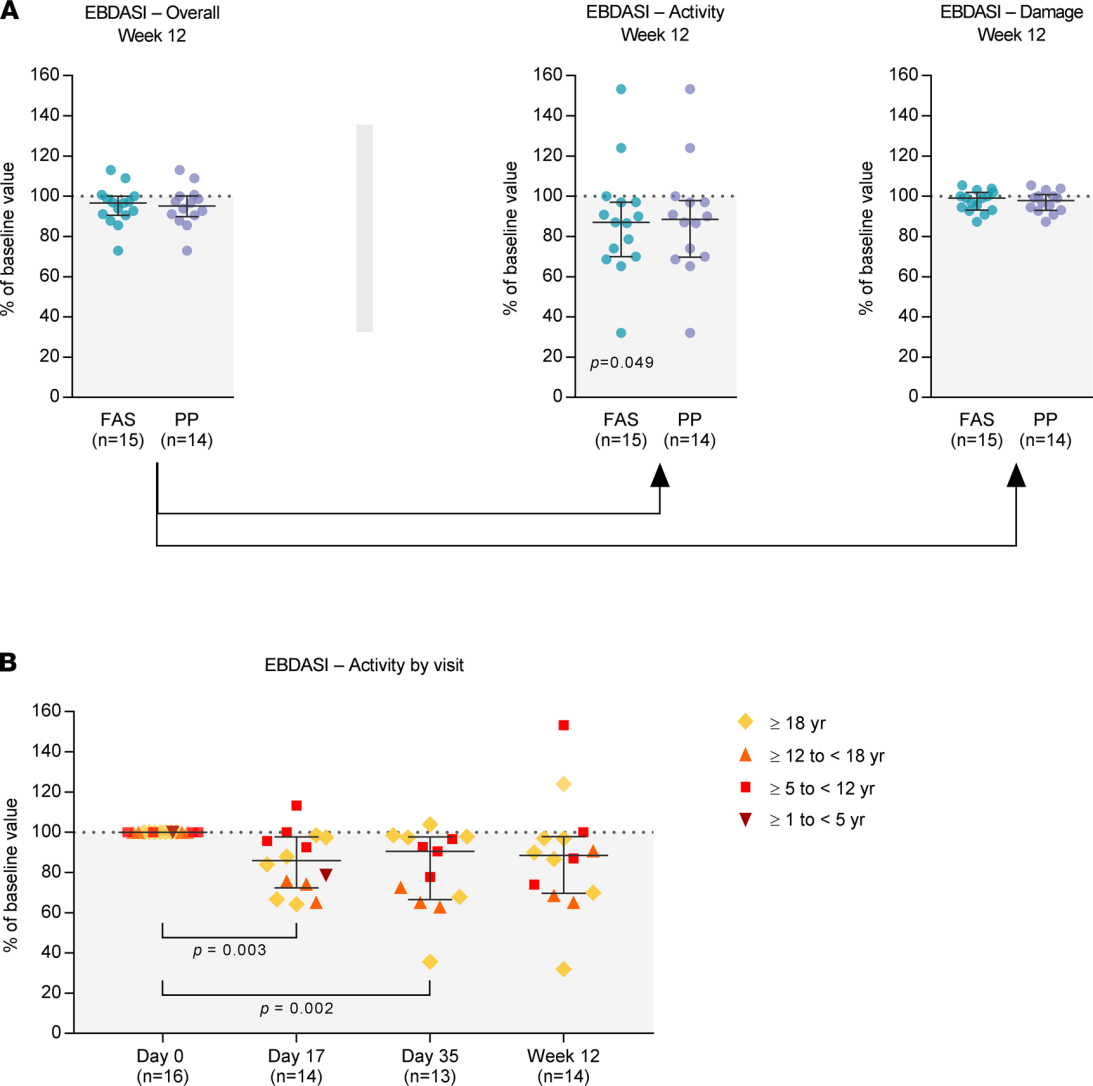
Changes in EBDASI. (**A**) Percent changes in the EBDASI overall score and total activity and damage subscores at 12 weeks (with the last observation carried forward [LOCF] in cases of missing data), expressed as percentage of the baseline value, in the full analysis set (FAS) and the per-protocol set (PP). (**B**) Percent changes in the EBDASI activity score by visit, expressed as percentage of the baseline value, in the FAS (no LOCF). Data are shown as medians with IQR; *P* values (2-sided Wilcoxon signed rank test) indicate statistical significance of changes from baseline. Kruskal-Wallis tests followed by Dunn’s multiple comparison tests revealed no statistically significant differences between the 3 postbaseline visits (day 17, day 35, and week 12; *P* > 0.05). For EBDASI overall and damage score data, see Supplemental Table 1.

During the 12‑week treatment and efficacy follow-up period, the median (IQR) Epidermolysis Bullosa Disease Activity and Scarring Index (EBDASI) (47) overall score decreased by 3.4% (0.0%–9.4%; FAS) and 4.8% (0.0%–9.4%; PP) as compared with baseline (Figure 3A), with the most pronounced changes occurring in cohort 2 (≥ 12 to < 18 years) (Supplemental Table 1; supplemental material available online with this article; https://doi.org/10.1172/jci.insight.151922DS1). Across all age cohorts, the observed change was mainly attributable to a decrease in the EBDASI activity subscore (median [IQR] reduction from baseline at week 12 of 13.0% [2.9%–30%; *P* = 0.049] in the FAS and 11.5% [2.9%–30%] in the PP), while the EBDASI damage subscore remained virtually unchanged (Figure 3A and Supplemental Table 1). The reduction of the EBDASI activity subscore was statistically significant already on days 17 and 35 (Figure 3B and Supplemental Table 1).

The percent change in the EBDASI activity subscore reflected a median (IQR) absolute reduction from baseline to week 12 of 5.5 (2.3–12.0) points, with 5 of 14 (36%) patients reaching or exceeding the minimal clinically important difference (MCID) defined for the EBDASI activity by Jain et al. as a decrease by ≥ 9 points (48) (Supplemental Figure 1, A–C). These patients reaching or exceeding the EBDASI activity MCID presented with a median (IQR) decrease in EBDASI activity from baseline of 31.4% (21.5%–51.3%) at week 12 (Supplemental Figure 1C).

### Changes in iscorEB scores.

**Figure 4:**
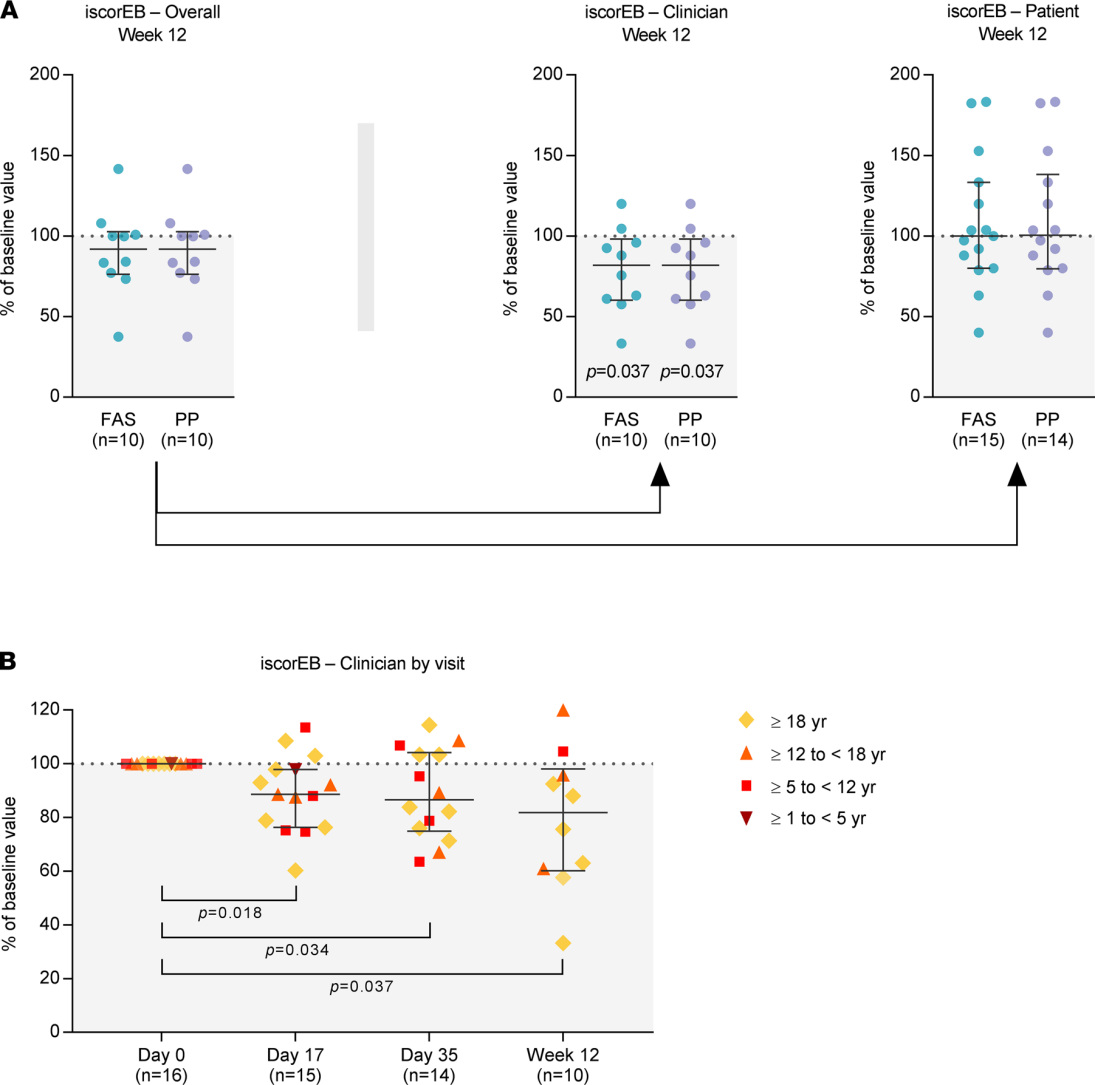
Changes in iscorEB. (**A**) Percent changes in the iscorEB overall score and iscorEB‑c and iscorEB‑p subscores at 12 weeks, expressed as percentage of the baseline value, in the full analysis set (FAS) and in the per-protocol set (PP). The lower number of data points for iscorEB overall and iscorEB‑c as compared with the iscorEB‑p is due to difficulties with blood sampling; for these patients, the lab values (anemia, albumin, inflammation) required for calculation of iscorEB overall and iscorEB‑c could not be obtained. (**B**) Percent changes in the iscorEB‑c score by visit, expressed as percentage of the baseline value, in the FAS. Data are shown as medians with IQR; *P* values (2‑sided Wilcoxon signed rank test) indicate statistical significance of changes from baseline. Kruskal-Wallis tests followed by Dunn’s multiple comparison tests revealed no statistically significant differences between the 3 postbaseline visits (day 17, day 35, and week 12; *P* > 0.05). For iscorEB overall and iscorEB‑p data, see Supplemental Table 2.

The median (IQR) Instrument for Scoring Clinical Outcome of Research for Epidermolysis Bullosa (iscorEB) (49) overall score decreased during the 12‑week treatment and efficacy follow-up period by 8.1% (–2.7% to 23.8%; FAS and PP) as compared with baseline (Figure 4A). Across all age cohorts, the observed change was mainly attributable to a statistically significant decrease in the clinician-reported section of the iscorEB (iscorEB‑c) (median [IQR] reduction from baseline at week 12 of 18.2% [1.9%–39.8%; *P =* 0.037] in the FAS and PP), while the patient-reported section of the iscorEB (iscorEB‑p) remained virtually unchanged (Figure 4A and Supplemental Table 2). The reduction in the iscorEB‑c subscore was already statistically significant on day 17. There seemed to be a trend toward further reduction of the iscorEB‑c with subsequent MSC infusions; however, the differences in the changes between the postbaseline visits were not statistically significant (Figure 4B and Supplemental Table 2).

The percent change in the iscorEB‑c score reflected an absolute median (IQR) reduction from baseline to week 12 of 6.7 (0.6–16.4) points, with 5 of 10 (50%) patients reaching or exceeding the MCID defined for the iscorEB‑c by Bruckner et al. as decrease by ≥ 5.5 points (50) (Supplemental Figure 1, D–F). These patients reaching or exceeding the iscorEB‑c MCID presented with a median (IQR) decrease in iscorEB‑c from baseline of 39.0% (30.7%–54.5%) at week 12 (Supplemental Figure 1F).

### Changes in itch and pain scores.

**Figure 5:**
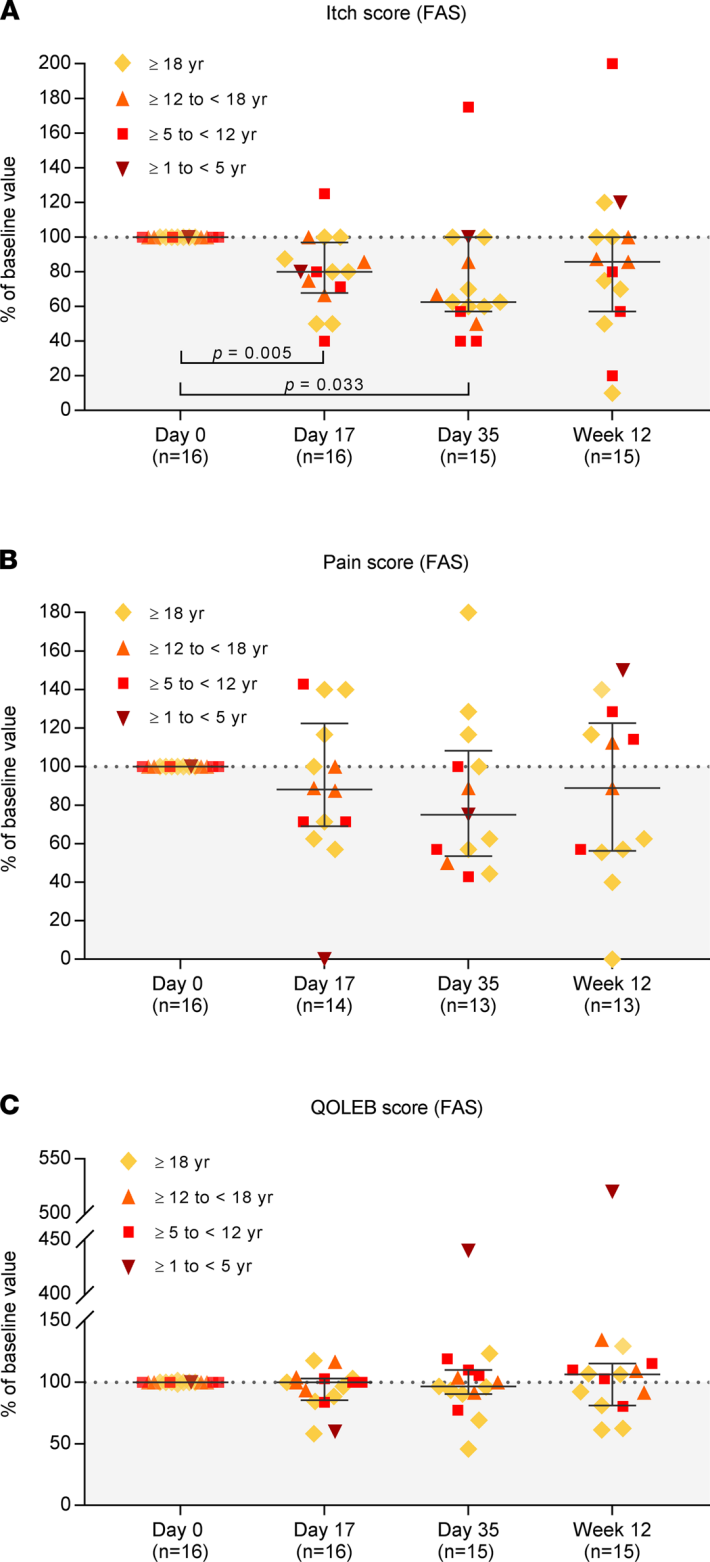
Changes in itch, pain, and impact of RDEB on life quality in the full analysis set (FAS). (**A**–**C**) Changes in: itch score, pain score, and QOLEB score, expressed as percentage of the baseline value. The lower number of data points for the pain score as compared with itch and QOLEB scores at the postbaseline visits (day 17, day 35, week 12) is caused by 2 patients presenting with pain score = 0 at baseline; therefore, for these patients, percent changes from baseline could not be calculated at any postbaseline visit. Please note that the patient who presented with an extreme percent increase in QOLEB score on day 35 and at week 12 (with score category changing from mild [day 0] to very mild [day 17] to moderate [day 35 and week 12]) had received only an incomplete second cell dose (day 17) and no third cell dose (day 35). Data are shown as medians with IQR; *P* values (2‑sided Wilcoxon signed rank test) indicate statistical significance of changes from baseline. Kruskal-Wallis tests followed by Dunn’s multiple comparison tests revealed no statistically significant differences between the 3 postbaseline visits (day 17, day 35, and week 12; *P* > 0.05). For the data of the per-protocol set, see Supplemental Figure 2.

During the treatment and efficacy follow-up period, median (IQR) itch score decreased from baseline by 20.0% (6.3%–31.0%), 37.5% (0%–42.9%), and 14.3% (0%–42.9%) in the FAS and by 20.0% (0.0%–33.5%), 37.5% (10.7%–44.6%), and 17.2% (0%–44.6%) in the PP on day 17, day 35, and week 12, respectively (Figure 5A and Supplemental Figure 2A). In all cohorts (except for cohort 4, where in 1 of 1 patient treatment was prematurely terminated at day 17), greatest reduction in itch score was reported on day 35 (Supplemental Table 3).

Median (IQR) pain score decreased from baseline by 11.8% (–22.5% to 30.8%), 25.0% (–8.4% to 46.4%), and 11.1% (–22.7% to 43.6%) in the FAS and by 11.8% (–34.2% to 28.6%), 24.3% (–12.5% to 48.2%), and 24.3% (–16.1% to 44.0%) in the PP on day 17, day 35, and week 12, respectively (Figure 5B and Supplemental Figure 2B). Cohort 1 reported the greatest pain score reduction at week 12, whereas in cohorts 2 and 3, greatest reduction in pain score was reported on day 35 (Supplemental Table 4).

### Changes in QOLEB scores.

The Quality of Life in Epidermolysis Bullosa (QOLEB) score (51) did not change substantially during the 12‑week treatment and efficacy follow-up period, as reflected by median changes from baseline of 0% (–14.4% to 3.0%), –3.4% (–9.4% to 10.0%), and 6.5% (–18.8% to 15.4%) in the FAS and 0% (–12.5% to 2.9%), –3.6% (–12.7% to 6.7%), and 4.7% (–18.9% to 11.4%) in the PP on day 17, day 35, and week 12, respectively (Figure 5C, Supplemental Figure 2C, and Supplemental Table 5).

### Serum HMGB1 concentrations.

**Figure 6:**
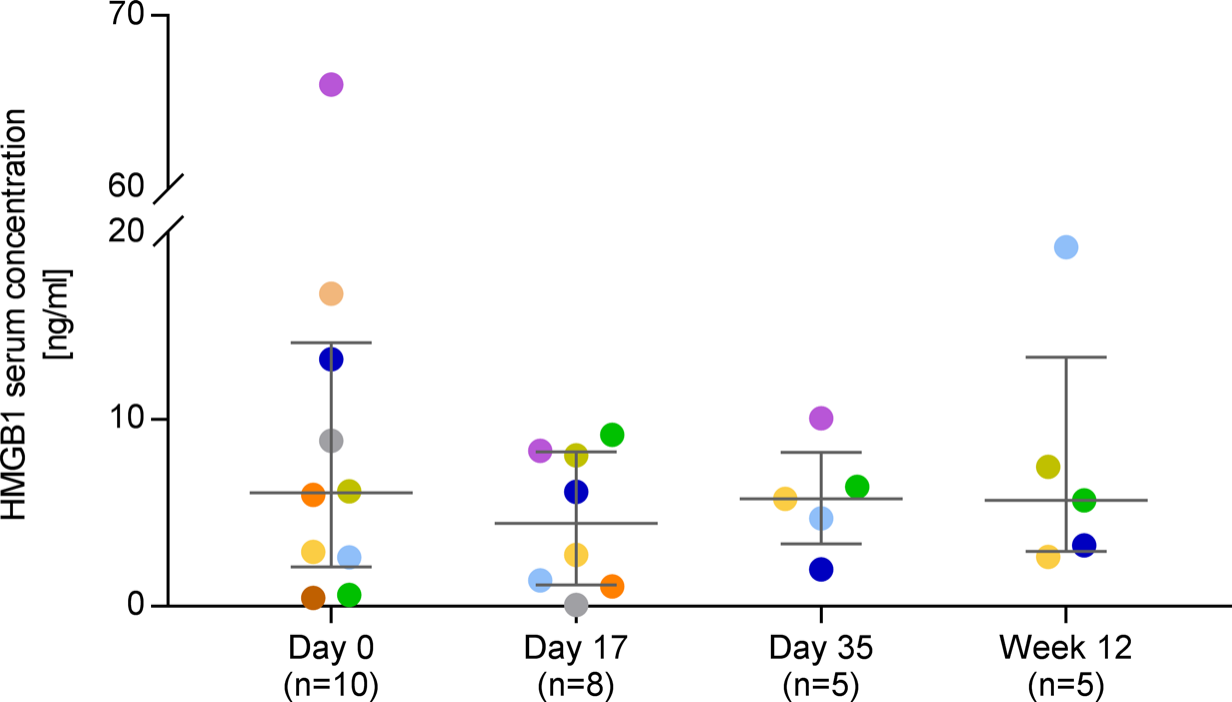
HMGB1 serum concentrations. Each color represents an individual patient. Data are shown as medians with IQR. Kruskal-Wallis tests followed by Dunn’s multiple comparison tests revealed no statistically significant differences between visits (*P* > 0.05).

Baseline high-mobility group box 1 (HMGB1) serum levels are available for *n* = 10 patients of cohorts 1 and 2 (≥ 18 years and ≥ 12 to < 18 years, respectively), and of these 10 patients, follow-up data are available for *n* = 8 (day 17) and *n* = 5 (day 35 and week 12). Baseline HMGB1 serum concentrations significantly correlated with disease severity, as measured by the EBDASI overall score (*r* = 0.709, *P* = 0.027; Supplemental Figure 3). Median HMGB1 serum concentrations (IQR) were 6.1 (2.1–14.1), 4.4 (1.1–8.3), 5.8 (3.3–8.3) and 5.7 (2.9–13.4) ng/mL on day 0, day 17, day 35, and week 12, respectively — without statistically significant differences between time points (Figure 6). All serum level values — except 1 HMGB1 serum level value — were below 20 ng/mL. Notably, in 1 patient in cohort 1 with a comparatively high baseline value of 66 ng/mL, HMGB1 serum level strongly decreased at the following visits (8 ng/mL and 10 ng/mL on days 17 and 35, respectively; week‑12 value not available) (Figure 6).

### Serum cytokine profiles.

Serum cytokine profiles covering 80 cytokines did not reveal any notable changes during the treatment and efficacy follow-up period (Supplemental Figure 4).

### Safety outcomes.

During the 12‑month safety follow-up, 69 treatment-emergent adverse events (TEAEs) were reported by 15 of 16 treated patients (Table 2). Most TEAEs were mild or moderate; 2 TEAEs (hypersensitivity) were severe. Three TEAEs, 1 mild lymphadenopathy and the 2 severe hypersensitivity events, were considered related to the cell product. The 2 hypersensitivity events were classified as serious, and the patients withdrew from further study treatment. Both serious events resolved on the day of onset; 1 patient stayed 1 night in the hospital for medical observation. All 3 product-related TEAEs resolved without sequelae. Beside the 2 hypersensitivity events and a transient mild decrease in blood pressure occurring during vital sign monitoring after the first cell infusion, no adverse events including infusion-related toxicities and changes in vital signs occurred during and within 2 hours after each infusion.

During the 12‑week treatment and efficacy follow-up period, no clinically relevant trend in vital signs occurred (Supplemental Table 6). Physical examination findings that were either not present at baseline or had changed from baseline were observed in 10 patients (Supplemental Table 7). Most changes affected the skin. Half of all changes represented improvements of preexisting conditions at baseline.

As required by the French National Agency for the Safety of Medicine and Health Products (ANSM), in order to assess the potential risk of alloimmunization, serum samples from the 2 patients enrolled in France were taken on day 17 and week 12 and subjected to anti-HLA antibody assessment (52). Anti-HLA antibodies were not detectable in either patient.

## Discussion

Although RDEB manifests at birth or in early childhood, several aspects of the disease develop, accumulate, and worsen during the patient’s lifetime. As long as no curative therapies are available, there is a desperate and urgent need for disease-modifying treatments that not only alleviate distressing symptoms, but also decelerate further accumulation of irreversible skin and organ damage. Therefore, in the present trial, the EBDASI was chosen as the primary efficacy endpoint because of its ability to distinguish ongoing disease activity (EBDASI activity), which would be responsive to disease-modifying therapy, from accumulative damage (EBDASI damage). The latter is not expected to improve as much with treatment (47) but can disclose whether and to what extent a treatment reduces or prevents further accumulation of damage (53). In addition, the iscorEB was recorded, which captures not only skin and mucosal, but also systemic clinician-reported EB complications (iscorEB‑c) alongside patient-reported perceptions of severity and impact (iscorEB‑p) (50).

A comparison study between the instruments EBDASI and iscorEB has detected a strong correlation between the subscores EBDASI activity and iscorEB‑c (53). The iscorEB was developed specifically for use in clinical trials with a predominant focus on disease activity as opposed to irreversible, permanent damage. In line with this study’s finding, we observed statistically significant decreases in both the EBDASI activity and the iscorEB‑c score (Figure 3A and Figure 4A). These reductions were already statistically significant on day 17 after the first cell infusion. While the EBDASI activity score remained on that level until week 12 (Figure 3B), the iscorEB‑c decreased further after the second and third cell infusion (Figure 4B). Thus, one may hypothesize that a further improvement could be achieved if the follow-up period was extended and/or additional cell infusions were administered. However, the observed differences in the iscorEB‑c changes between the postbaseline visits were not statistically significant, and further studies covering a longer efficacy follow-up period remain necessary to confirm this hypothesis.

To evaluate the clinical significance of the present findings about disease activity, we compared the absolute changes to the MCIDs determined by Jain et al. (48) for EBDASI activity subscale and Bruckner et al. (50) for iscorEB‑c. Applying their thresholds, 36% and 50% of patients experienced a clinically meaningful improvement of the disease during the 12‑week treatment and efficacy follow-up period as measured by EBDASI activity and iscorEB‑c, respectively (Supplemental Figure 1, A and D). The patients who showed a clinically meaningful response to ABCB5^+^ MSC treatment achieved median decreases in EBDASI activity and iscorEB‑c from baseline at week 12 as high as 31.4% and 39.0%, respectively (Supplemental Figure 1, C and F).

Since RDEB is a progressive disorder, the clinically meaningful improvement in disease activity observed in these patients may, beyond current symptom relief, be beneficial for prevention of future RDEB-related cutaneous and systemic manifestations. In line with this, while disease activity obviously decreased following infusions with ABCB5^+^ MSCs, the EBDASI damage score remained unchanged (Figure 3A), indicating that no further damage had accumulated during efficacy follow-up. It might be speculated that the observed reduction in disease activity could have contributed to a deceleration in damage accumulation; however, this remains to be investigated in a controlled trial examining a longer follow-up period.

The observed decrease in disease activity was reflected by alleviations of pruritus and pain (Figure 5, A and B, and Supplemental Figure 2, A and B), which represent 2 of the most distressing symptoms creating physical, psychological, and social burdens on the everyday lives of patients with RDEB (54–59). It may seem striking that the median reduction in both scores was greatest at day 35, which might be attributed to the shorter interval between the day 35 visit and the preceding (second) cell infusion (18 days) as compared with the week 12 visit and the preceding (third) cell infusion (49 days). However, the differences in the changes between the postbaseline visits were not statistically significant, and further studies remain necessary to identify the optimum application frequency and interval of the cell infusions.

As with EBDASI and iscorEB, the question arises whether the changes in itch score are not only statistically significant, but also clinically meaningful. However, MCIDs for itch scores in RDEB have, to our knowledge, not yet been reported. Itch is consistently rated as the most troublesome symptom in severe types of epidermolysis bullosa, such as RDEB, entailing not only a physical, but also psychological and social burden on the patients’ everyday life (56–59). Therapies that can provide effective pruritus relief are highly needed (58, 59). Thus, while we clearly see statistically significant reductions in itch score, it would be important to have substantiated RDEB-specific target values to enable a patient-centered evaluation of the therapeutic efficacy of ABCB5^+^ MSCs and other new treatments.

It may seem astonishing that the observed improvements in EBDASI activity, iscorEB‑c, and itch scores did not translate into an improvement in the QOLEB score (Figure 5C and Supplemental Figure 2C). However, the QOLEB incorporates a range of aspects related to disease damage — e.g., ability to move, ability to write, and ability to eat (51). Accordingly, the QOLEB score was found to strongly correlate with the EBDASI damage and iscorEB‑p but not EBDASI activity and iscorEB‑c scores (47, 50, 53). This suggests that the QOLEB captures a large part of the effects on quality of life that are more related to accumulated damage, such as flexion contractures, pseudosyndactyly strictures, or esophageal strictures, rather than to fluctuating, modifiable disease activity (47). A longer follow-up would probably be required to detect potential changes in the QOLEB score. Furthermore, a greater impact on life quality might be apparent in younger patients who have not yet accumulated irreversible damage resistant to impact from short-term decreases in disease activity. Nevertheless, symptoms of disease activity, even if potentially underestimated by the QOLEB, substantially impact the quality of life of patients with RDEB (54–59). Among these, untreatable pruritus is associated with a particularly great burden on the life quality of patients with RDEB, as it not only triggers an itch-scratch cycle that promotes blister formation, deteriorates existing wounds, and potentially increases the risk of infections, but it also is extremely bothersome to patients and disturbs their sleep (56–59). Thus, it might be conceivable that the reduction in itch observed in the present study has improved life quality, even though this was not captured by the QOLEB score.

The alarmin HMGB1 has been suggested as a serum biomarker of RDEB disease severity, since levels positively correlate with the extent of skin blistering in RDEB (60). In the present study, except for a nonsignificant trend toward decrease from baseline to day 17 (from median 6.1 to 4.4 ng/mL), we did not observe consistent overall reductions in HMGB1 serum levels (Figure 6). Observed decreases in HMGB1 serum levels during MSC treatment were more pronounced in the patients with higher baseline levels (Figure 6), which was also seen in a recent study by Rashidghamat et al. (31) of i.v. infusions of BM-derived MSCs to treat RDEB. Thus, in the present study, detection of a potential overall treatment effect on HMGB1 levels might have been hampered by the generally low HMGB1 baseline levels (median 6.1 ng/mL). These were strikingly lower than previously reported by Petrof et al. (60) for patients with RDEB (median 21.0 ng/mL), which might reflect the younger patient population in the present study (4–36 years versus 17–88 years). This is supported by the study of Rashidghamat et al. (31) in a patient population whose age span (26–55 years) ranged between the present study and that of Petrof et al. (60) In these patients, also the median basal HMGB1 level (approximately 8 ng/mL; value deduced from Figure 3 of the publication; ref. 31) was between the present and the Petrof trial (60). Since HMGB1 is released upon cellular stress or cell death — not only from skin, but all cell types (61, 62) — it may be expected that elevations in HMGB1 levels in RDEB over healthy subjects become more pronounced with age in parallel with accumulation of extracutaneous manifestations. Furthermore, HMGB1 serum levels were found to positively correlate with disease severity as captured by the Birmingham Epidermolysis Bullosa Severity Score (60) and the EBDASI (Supplemental Figure 3), which in turn are reported to increase with age (48, 63). Thus, robust reference values from age-matched healthy controls are needed for further exploration of HMGB1 as a disease severity marker in RDEB. In addition, there may be intraindividual variations and fluctuations in circulating HMGB1 levels in patients with RDEB, and these remain to be elucidated, as well.

In view of the high disease burden and the urgent medical need of patients with RDEB, it would be desirable to distinguish patients who are likely to benefit from a specific treatment from those who are not. Basically, for MSC therapy approaches, it has been observed across a broad range of diseases that a certain proportion of patients do not respond to treatment (64). While a major part of observed variabilities in clinical outcomes following MSC therapy has been ascribed to heterogenous products with insufficiently characterized therapeutic potency activity (65), the thoroughly standardized quality and biological activity of the present cell product (Supplemental Table 8) counts against potential differences in product quality as a cause of variation in the treatment responses. Apart from product-related factors, certain patient-individual characteristics may determine the responsiveness to treatment, suggesting that the detection of characteristic biomarkers or genetics could enable better prediction of treatment efficacy (64, 66). While, in the present study, there was no correlation between baseline levels of any of the cytokines studied and the change in disease severity scores after the cell infusions, it remains to be elucidated whether the response to ABCB5^+^ MSC treatment might be associated with the patient’s genotype.

In general, treatment with ABCB5^+^ MSCs was well tolerated, with only 3 TEAEs being considered related to the cell product. Two of these were severe hypersensitivity reactions, classified as serious. However, these events were manageable and recovered without sequelae on the day of onset. In general, hypersensitivity reactions to intravascular infusion of unmatched allogeneic MSCs have only very rarely been reported (67, 68). In the light of evidence suggesting that allogeneic MSCs can induce immune responses (69, 70), it seems conceivable that the events observed in the present study might have resulted from immunological sensitization, even though the 2 affected patients had not been tested for anti-HLA antibodies. Studies evaluating potential sensitization by i.v. allogeneic MSCs have revealed that between 0% and 19% of patients developed donor-specific anti-HLA antibodies; however, this was not associated with any clinically apparent adverse events (71–73). In addition, ABCB5^+^ MSCs do not express the MHC class II surface receptor HLA‑DR (TICEBA, unpublished data). Apart from HLA sensitization, nonimmunological reactions to product-related factors have been discussed as potential causes of hypersensitivity reactions in MSC therapy, including residual DMSO used for cryopreservation (74, 75) and impurities resulting from necrotic cells (67). However, the standardized and strictly controlled manufacturing process of the ABCB5^+^ MSC product ensures effective DMSO depletion and a consistently high viability and vitality of the cells (45) (Supplemental Table 8), which argues against product impurities as elicitors of hypersensitivity. In very rare cases, hypersensitivity reactions to HSA, an excipient of the vehicle solution, have been reported, with a documented rate of less than 0.1% (76).

While the cause of the 2 hypersensitivity events remains unclear, premedication with antihistamines could decrease the risk of hypersensitivity. In the present trial, premedication was not required by the protocol, though it was used on an institution-by-institution basis. Notably the 2 patients who experienced hypersensitivity reactions were not premedicated. Overall, the Trial Data Monitoring Committee evaluated the potential risk of hypersensitivity reactions as being justified by the anticipated benefits of treatment with ABCB5^+^ MSCs for patients with RDEB, and the committee recommended premedication with antihistamines to minimize the risk in the future. In addition, for a subsequent study, we are planning to systematically monitor potential induction of anti-HLA antibodies in all patients and, in cases of hypersensitivity events, perform T cell proliferation assays to discriminate between immunological and nonimmunological reactions.

Naturally, the present study is limited by factors typically associated with early-phase trials and particularly with orphan indications, including a small number of patients and an open, noncomparative design. Patients could have benefitted from the additional care they might have received during the trial. In addition, and equally important, there may be natural fluctuations in the disease status that could not be ascertained during the comparatively short efficacy follow-up period, as well as patient-specific factors affecting the course of disease; we did not control for either of these. Despite these limitations, we conclude that i.v. therapy with ABCB5^+^ MSCs might deliver clinical benefit to patients, including reduction of disease activity and alleviation of the 2 most common and bothersome symptoms: itch and pain. Furthermore, beyond potential disease activity–related improvements, it might be anticipated that repeated doses given over a longer period of time could, by decreasing disease activity, reduce further damage accumulation. Interestingly, very recently, it was shown that ABCB5^+^ MSCs possess a superior homing potential to injured tissues compared with BM-derived MSCs, presumably due to increased *HOXA3* gene expression, and are capable of secreting type VII collagen (77). Thus, it might be speculated that long-term treatment with ABCB5^+^ MSCs, beyond alleviating disease activity, could even enhance skin and mucosal structural integrity via accumulated deposition of type VII collagen. In this perspective, it may be important to start the treatment early in life, before the onset of functional damage or other complications. Taken together, we regard the present findings as providing a rationale for conducting a larger and longer-term trial with multiple infusions and a randomized, placebo-controlled design together with refined outcome parameters, including determination of type VII collagen deposition in skin biopsies, to confirm potential benefit, optimize the dosing regimen, and evaluate the long-term efficacy and safety of the therapy. A longer efficacy follow-up would also enable us to ascertain natural fluctuations in disease activity and to investigate the duration of benefit. At present, the ATMP, referred to as allo-APZ2-EB, has been granted Orphan Drug Designation by the US Food and Drug Administration and the European Medicines Agency.

## Methods

### Patients.

Patients (1–55 years) were eligible if they were diagnosed with RDEB by genotypic (mutation analysis) and phenotypic (wound assessment) evaluation and had a negative salt-split skin immunofluorescence test for antibasement membrane zone antibodies. Main exclusion criteria included the following: previous or current cancer, impaired pulmonary or cardiovascular function, history or risk of thrombosis, clinically significant or unstable comorbidities, or any other condition that might interfere with the trial treatment, affect the patient’s compliance, or confer a risk of treatment-related complications to the patient.

### Trial design.

The study was an international (Germany, Austria, France, United Kingdom, and USA), multicentric, single-arm, open-label, phase I/IIa trial consisting of 3 periods: screening (1 week), treatment and efficacy follow-up (day 0 to week 12), and safety follow-up (until end of month 12) (Figure 1A). Patients were successively enrolled into 4 age cohorts (Figure 1B). For each cohort, at least 3 patients needed to be treated and followed up for 2 weeks following the third cell application (cohort 1) or first cell application (subsequent cohorts), and their safety data needed to be evaluated by a Data Monitoring Committee applying predefined stopping rules before the next cohort could be opened.

### Interventions.

Donor-derived, ex vivo expanded ABCB5^+^ MSCs were delivered as Good Manufacturing Practice–conforming (GMP-conforming) standardized ATMP of proven vitality, viability, and biological activity (potency) (see Supplemental Table 8 for product release data). Patients received 3 i.v. infusions of 2 × 10^6^ allogeneic ABCB5^+^ MSCs/kg body weight suspended in Ringer’s lactate solution containing 2.5% HSA and 0.4% glucose at a concentration of 1 × 10^7^ cells/mL, infused at a rate of 1–2 mL/min on days 0, 17, and 35. Patients were monitored for at least 2 hours after infusion, and vital signs — including respiratory frequency, heart rate, blood pressure, body temperature, and oxygen saturation — were recorded prior and at 10–15 minutes, 20–30 minutes, 1 hour, and 2 hours after infusion.

### Outcome measures.

The primary efficacy end point was overall improvement of EB symptoms at 12 weeks, measured as a percent change of the EBDASI (overall, total activity, and total damage scores) from baseline, with last observation carried forward (LOCF) in case of missing week 12 values.

Secondary efficacy endpoints were percent change of the EBDASI (overall, total activity, and total damage) (47); iscorEB (overall, iscorEB‑c, and iscorEB‑p) (49); itch, pain, and QOLEB (51) scores from baseline on day 17, day 35, and week 12; and serum inflammation markers (HMGB1 levels and cytokine profile) on days 0, 17, and 35 and at week 12.

Itch and pain were assessed using 0- to 10‑point numerical rating scales with 0 representing no pain and 10 representing worst imaginable itch/pain. Serum HMGB1 levels were measured by using a sandwich ELISA developed by Shino-Test Corporation, Tokyo, Japan (ST51011, purchased from IBL International; intraassay CV 5.5%–13.7%, interassay CV 7.6%–13.7%, as per manufacturer’s information) according to the manufacturer’s instructions. Serum cytokine profiles were determined using an antibody pair-based chemiluminescent assay simultaneously detecting 80 human cytokines (Human Cytokine Antibody Array — Membrane, ab133998, Abcam) according to the manufacturer’s instructions. Signal densities were spotted from digitized images, corrected for background density and normalized against positive control signals using ImageJ software (NIH).

Primary safety outcome was the occurrence of adverse events during the 12‑month safety follow-up. Secondary safety outcomes were vital signs and physical examination findings during the 12‑week treatment and efficacy follow-up period. In the patients enrolled in France, serum samples taken on day 17 and at week 12 were subjected to anti-HLA antibody assessment by solid-phase assays on an Immucor instrument using the Lifescreen Deluxe (LMX) kit for screening and the Luminex Single Antigen Assays I and II for specification (all from Immucor).

### Statistics.

Planned sample size was 16 patients. Efficacy analyses were performed on the FAS, which included all patients who received at least 1 cell dose (*n* = 16), and on the PP, which included all patients of the FAS who had no major (defined as potentially influencing efficacy results) protocol deviations (*n* = 14). The safety analysis set was identical to the FAS (*n* = 16). Statistical analyses were performed using GraphPad Prism 7 software (GraphPad Software). Statistical significance of median percent changes from baseline was tested against the null hypothesis (median percent change = 0) using a 2‑sided Wilcoxon signed rank test. Statistical significance of differences between the different time points was tested by Kruskal-Wallis tests, followed by Dunn’s multiple comparisons tests. Spearman’s rank correlation analyses were performed to test for associations between variables.

### Study approval.

The trial complied with the principles of the Helsinki Declaration and Good Clinical Practice. The study protocol and all other relevant documents had been approved by the competent drug regulatory authorities and the appropriate local independent ethics committees/institutional review boards: Ethics Committee of the Albert Ludwig University of Freiburg, Freiburg, Germany; Ethics Committee for the State of Salzburg, Salzburg, Austria; Comité de Protection des Personnes (CPP) Ile de France 8, Boulogne-Billancourt, France; Ethics Committee at the Ospedale Pediatrico Bambino Gesù, Roma, Italy; North East — York Research Ethics Committee, Newcastle upon Tyne, United Kingdom; and University of Minnesota Institutional Review Board, Minneapolis, Minnesota, USA. Prior to any trial-related activities/procedures, all patients gave written informed consent. All patient photographs contained in this report are used with the patients’ approval.

## Author contributions

ENR wrote the manuscript. DK, KD, CLE, JT, and MAK contributed to protocol design. DK, KD, SF, CD, FS, SG, ML, JWB, AH, GZ, MEH, EB, MP, GP, SK, CLE, AEM, JAM, and JT undertook the clinical trial and contributed to data acquisition, analysis, and interpretation. KD, ENR, SF, CD, and MAK analyzed data. DK, KD, SF, CD, LE, and MAK performed diagnostic studies and analysis. JE, SS, SB, LE, CG, and MAK contributed to the manufacture of the IMP (GMP-grade ABCB5^+^ MSCs). DK and JT were members of the trial safety committee. MHF, NYF, and MAK provided stem cell–related scientific and clinical advice and support. All authors contributed to manuscript review and approval of the final version. DK, KD, and ENR contributed equally. DK is listed first because she was the coordinating investigator of this clinical trial. JT and MAK contributed equally. MAK is listed last since he supervised the whole trial and the data analysis.

## Supplementary Material

Supplemental data

ICMJE disclosure forms

## Figures and Tables

**Table 1:**
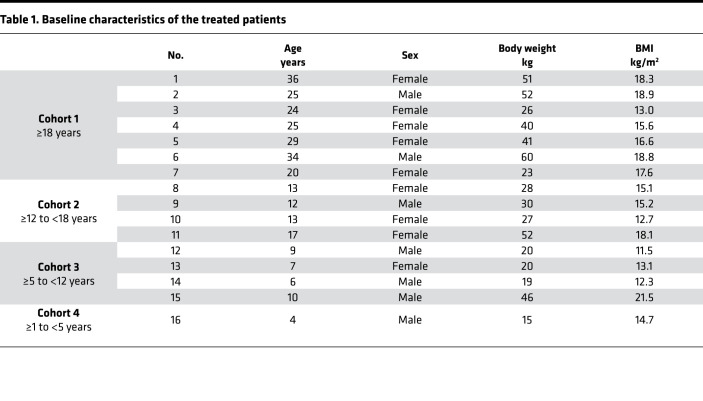
Baseline characteristics of the treated patients

**Table 2:**
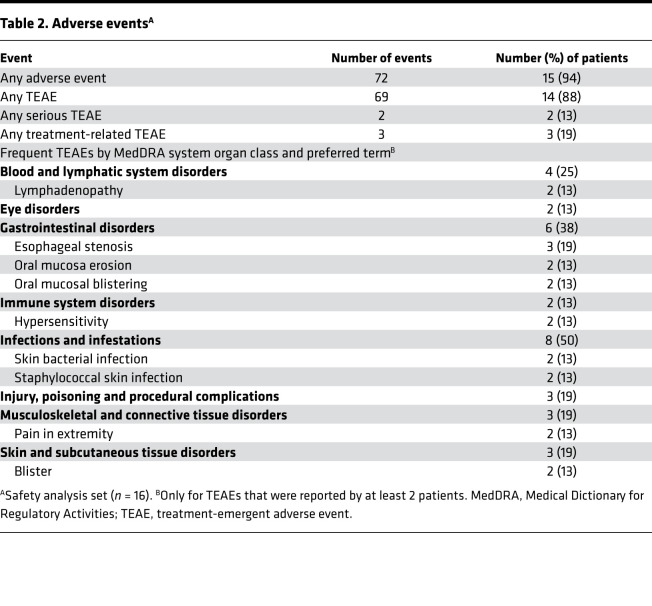
Adverse events^A^

## Version 1 10/19/2021

In-Press Preview

## Version 2 11/22/2021

Electronic publication
