# Supplementary material for: Clinical trial of ABCB5+ mesenchymal stem cells for recessive dystrophic epidermolysis bullosa
Source: JCI Insight. 2021 Nov 22;6(22):e151922. doi: 10.1172/jci.insight.151922 (PMC8663784; doi:10.1172/jci.insight.151922)
Supplement: Supplemental data [file jciinsight-6-151922-s055.pdf]

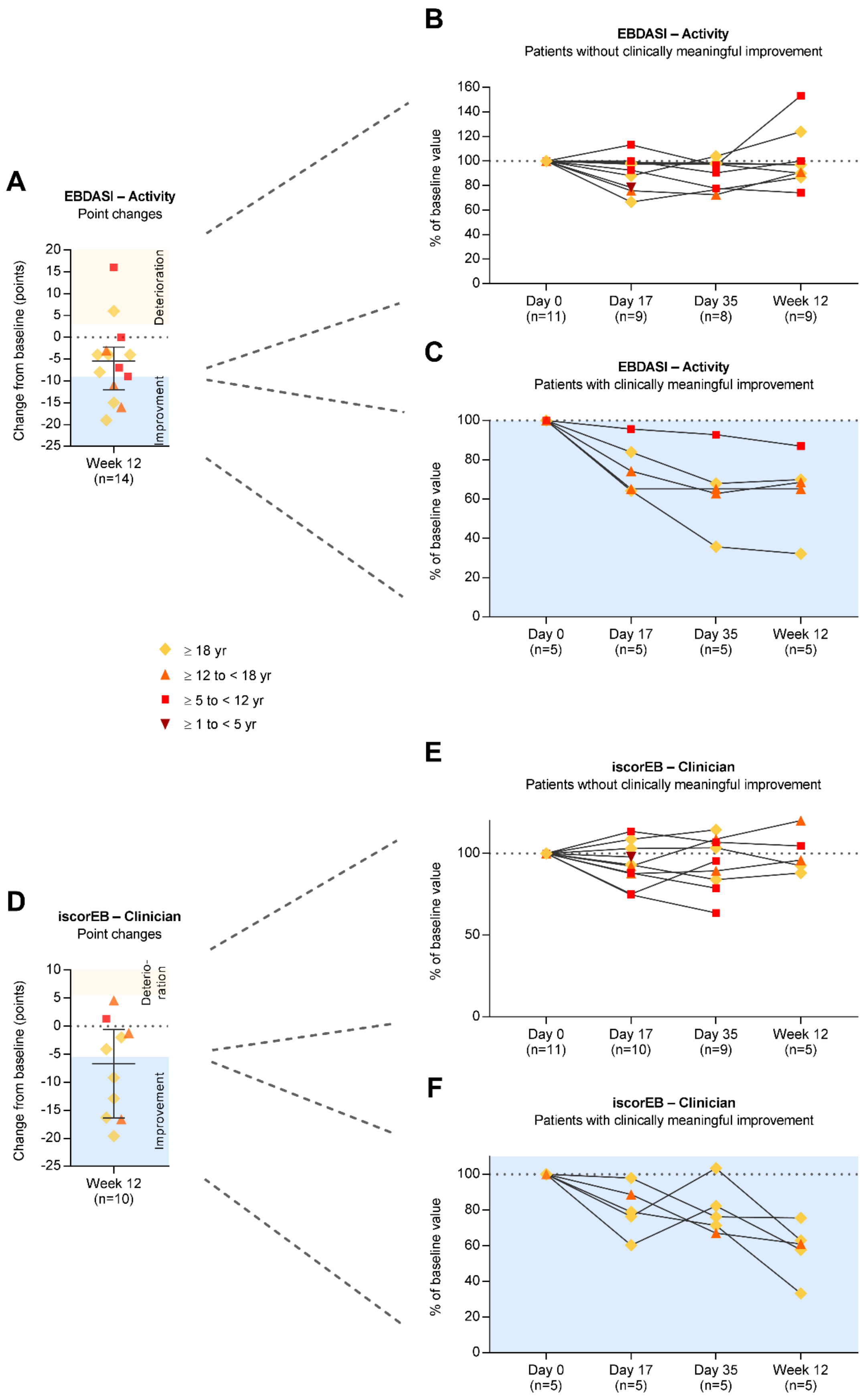

Supplemental Figure 1. For legend see next page.

Supplemental Figure 1. Changes in EBDASI *activity* and *iscorEB-c* in patients without and with clinically meaningful disease improvement. (**A**) Absolute changes in the EBDASI *activity* score from baseline at 12 weeks. Colored areas capture values reaching or exceeding the MCID for disease improvement (≥9 points decrease, blue) and deterioration (≥3 points increase, yellow). Error bar shows median with IQR. (**B, C**) Percent changes in the EBDASI *activity* score by visit in all patients without (B) and with (C) clinically meaningful disease improvement as shown in (A). (**D**) Absolute changes in the *iscorEB-c* score from baseline at 12 weeks. Colored areas capture values reaching or exceeding the MCID for disease improvement (≥5.5 points decrease, blue) and deterioration (≥5.5 points increase, yellow). Error bar shows median with IQR. (**E, F**) Percent changes in the *iscorEB-c* score by visit in all patients without (E) and with (F) clinically meaningful disease improvement as shown in (D).

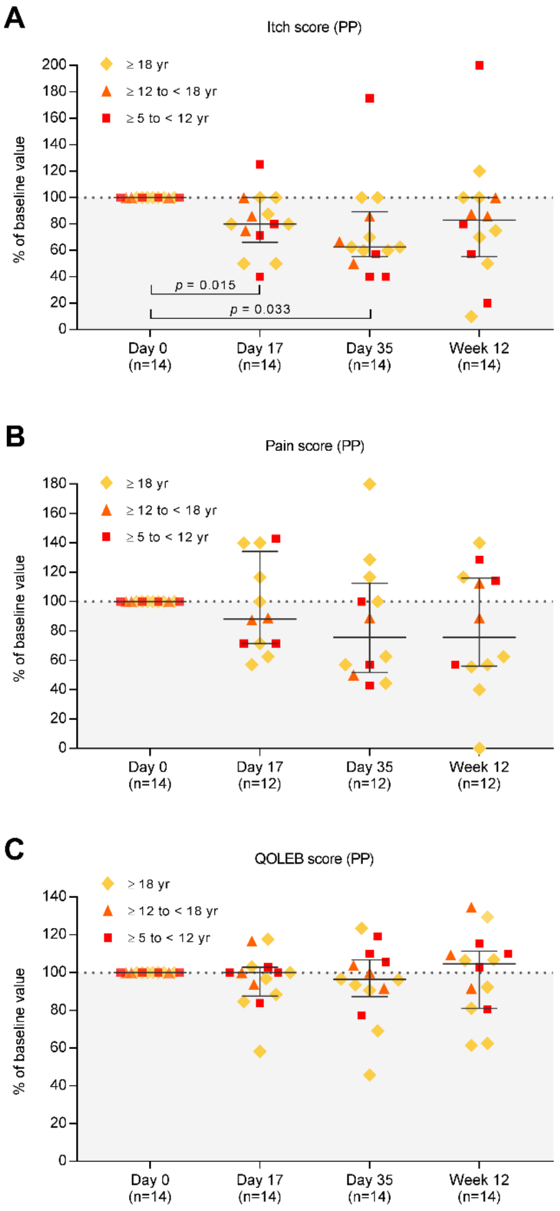

Supplemental Figure 2. Changes in itch, pain and impact of RDEB on life quality in the per-protocol set (PP). Changes in: (**A**) itch score, (**B**) pain score, and (**C**) QOLEB score, expressed as percentage of the baseline value. The lower number of data points for the pain score as compared to itch and QOLEB scores at the post-baseline visits (day 17, day 35, week 12) is caused by 2 patients presenting with pain score = 0 at baseline, so that for these patients percent changes from baseline could not be calculated at any post-baseline visit. Error bars show medians with IQR; *p* values (two-sided Wilcoxon signed rank test) indicate statistical significance of changes from baseline. Kruskal-Wallis tests followed by Dunn’s multiple comparison tests revealed no statistically significant differences between the three post-baseline visits (day 17, day 35 and week 12; $p>0.05$). For the data of the full analysis set Figure 5.

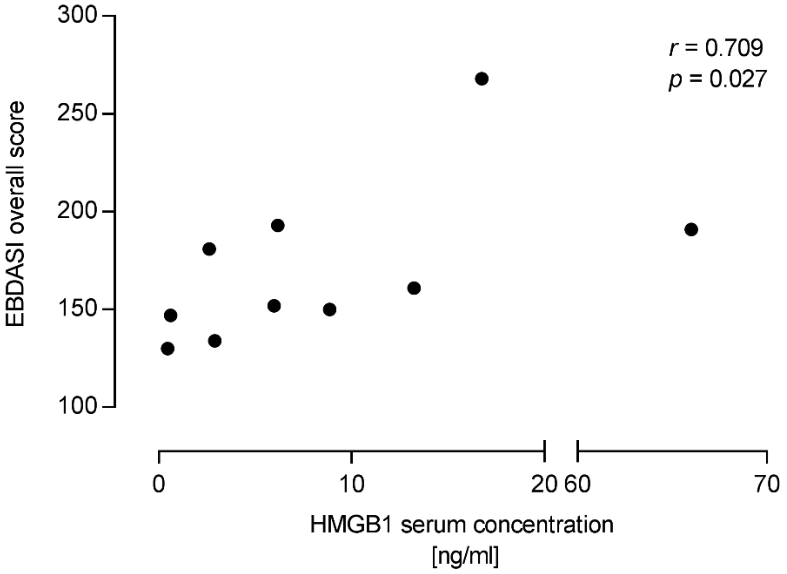

Supplemental Figure 3. Spearman's rank correlation analysis showing a significant association between baseline HMGB1 serum concentrations and EBDASI overall score (n=10).

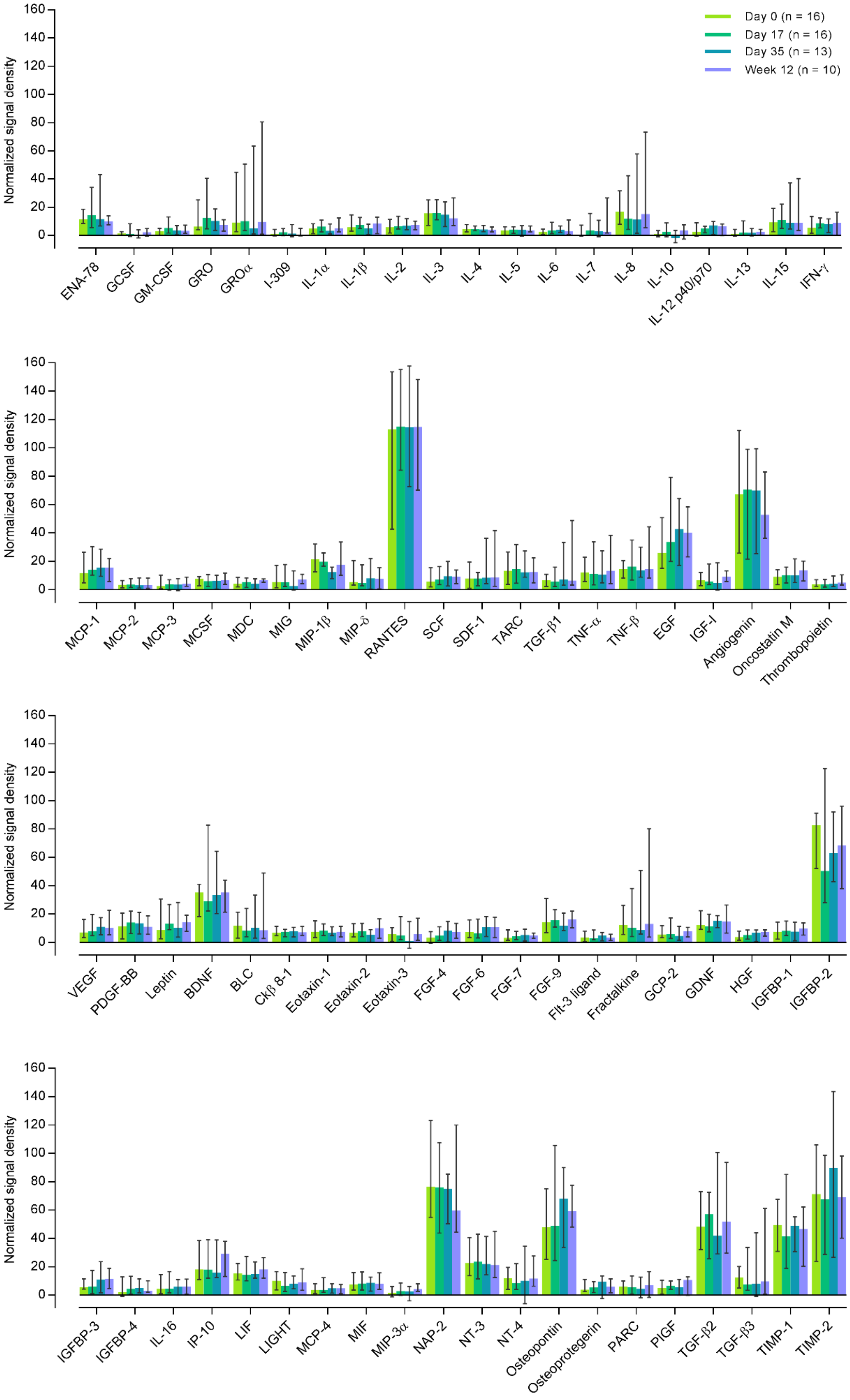

Supplemental Figure 4. For legend see next page.

Supplemental Figure 4. Serum cytokine profiles as determined by cytokine antibody array. Shown are medians with IQR of 16 (day 0), 16 (day 17), 13 (day 35) and 10 (week 12) patients. BDNF, Brain-derived neurotrophic factor; BLC, B lymphocyte chemoattractant; ENA, Epithelial-derived neutrophil-activating peptide; Flt-3, Fms-like tyrosine kinase; GCP, Granulocyte chemotactic protein; GCSF, Granulocyte colony-stimulating factor; GDNF, Glial cell line-derived neurotrophic factor; GRO, Growth-regulated oncogene; IGFBP, IGF-binding protein; IP, IFN-γ-induced protein; LIF, Leukemia inhibitory factor; LIGHT, Homologous to **l**ymphotoxin, exhibits **i**nducible expression and competes with HSV **g**lycoprotein D for binding to **h**erpesvirus entry mediator, a receptor expressed on **T** lymphocytes (CD258); MCP, monocyte chemoattractant protein; MCSF, Macrophage colony-stimulating factor; MDC, macrophage-derived chemokine; MIF, Macrophage migration inhibitory factor; MIP, Macrophage inflammatory protein; NAP, Neutrophil-activating peptide; NT, neurotrophin; PARC, Pulmonary and activation-regulated chemokine; PIGF, Placental growth factor; SCF, stem cell factor; SDF, Stromal cell-derived factor; TARC, Thymus and activation-regulated chemokine; TIMP, Tissue inhibitor of metalloproteinases.

Supplemental Table 1. Percent change in EBDASI scores from baseline by age cohort and visit[A]

Score	Visit	Cohort 1 (≥18 yr) N = 7 (FAS & PP)	Cohort 2 (≥12 to <18 yr) N = 4 (FAS)/N = 3 (PP)[B]	Cohort 3 (≥5 to <12 yr) N = 4 (FAS & PP)	Cohort 4 (≥1 to <5 yr) N = 1 (FAS only)	All patients N = 16 (FAS)	All patients N = 14 (PP)
Overall	Day 17	-5.0 (-12.0; 0.4)[C]	-9.9 (-11.8; -5.2)[D]	-2.3 (-0.6; 3.2)	-3.1	-2.9 (-9.9; 1.5)[E]	-2.6 (-9.9; 1.5)[C]
	Day 35	-1,4 (-19.7; 0.7)[C]	-11.6 (-11.6; -10.6)[D]	1.5 (-3.1; 4.3)	–[C]	-1.4 (-11.6; 1.0)[F]	-1.4 (-11.6; 1.0)[C]
	Week 12	-3.4 (-14.5; 0.7)	-9.4 (-12.2; -6.2)[D]	-1.3 (-4.9; 6.6)	–[C]	-4.8 (-9.4; 0.0)[E]	-4.8 (-9.4; 0.0)
	Week 12 (LOCF)	-3.4 (-14.5; 0.7)	-9.4 (-12.2; -6.2)[D]	-1.3 (-4.9; 6.6)	-3.1	-3.4 (-9.4; 0.0)[C]	-4.8 (-9.4; 0.0)
Activity	Day 17	-14.0 (-33.3; -2.5)[C]	-25.7 (-34.8; -24.2)[D]	-2.2 (-5.9; 6.7)	-21.4	-14.0 (-25.7; -2.5)[E]	-12.0 (-25.7; -2.5)[C]
	Day 35	-2.3 (-32.0; -1.5)[C]	-34.8 (-37.1; -27.3)[D]	-8.4 (-15.9; -5.3)	–[C]	-9.5 (-32.0; -2.5)[F]	-9.5 (-32.0; -2.5)[C]
	Week 12	-10.0 (-30.0; -2.9)	-31.4 (-34.8; -9.1)[D]	-6.5 (-19.5; 26.7)	–[C]	-11.5 (-30.0; -2.9)[E]	-11.5 (-30.0; -2.9)
	Week 12 (LOCF)	-10.0 (-30.0; -2.9)	-31.4 (-34.8; -9.1)[D]	-6.5 (-19.5; 26.7)	-21.4	-13.0 (-30.0; -2.9)[C]	-11.5 (-30.0; -2.9)
Damage	Day 17	1.2 (-6.3; 2.2)[C]	-1.5 (-8.6; 0.0)[D]	2.7 (-0.4; 4.7)	2.0	0.0 (-2.3; 2.2)[E]	0.0 (-2.3; 2.2)[C]
	Day 35	-0.6 (-12.7; 4.6)	-5.1 (-6.3; -3.7)[D]	4.4 (-0.5; 9.3)	–[C]	-0.8 (-5.1; 4.6)[E]	-0.8 (-5.1; 4.6)
	Week 12	-0.9 (-9.1; 4.4)	-5.5 (-7.3; -0.7)[D]	1.6 (1.6; 3.5)	–[C]	-0.8 (-6.9; 3.2)[E]	-0.8 (-6.9; 3.2)
	Week 12 (LOCF)	-0.9 (-9.1; 4.4)	-5.5 (-7.3; -0.7)[D]	1.6 (1.6; 3.5)	2.0	-0.7 (-6.9; 3.2)[C]	-0.8 (-6.9; 3.2)

[A]Data are medians (IQR). [B]Values are identical for the full analysis set (FAS) and per-protocol set (PP), because the patient that was excluded from the PP had no post-baseline measurement (which is why the patient's values are not contained in the FAS either). [C]Value from 1 patient missing. [D]Value from 1 patient (FAS) missing. [E]Values from 2 patients missing. [F]Values from 3 patients missing.

Supplemental Table 2. Percent change in iscorEB scores from baseline by age cohort and visit[A]

Score	Visit	Cohort 1 (≥18 yr) N = 7 (FAS & PP)	Cohort 2 (≥12 to <18 yr) N = 4 (FAS)/N = 3 (PP)[B]	Cohort 3 (≥5 to <12 yr) N = 4 (FAS & PP)	Cohort 4 (≥1 to <5 yr) N = 1 (FAS only)	All patients	
						N = 16 (FAS)	N = 14 (PP)
Overall	Day 17	-8.5 (-15.4; -0.2)	-9.3 (-18.4; 17.7)[D]	-17.3 (-28.5; -5.1)	-30.7	-9.3 (-22.1; -2.6)[C]	-9.1 (-19.3; -2.0)
	Day 35	-2.0 (-6.8; 1.3)	-8.4 (-29.2; 40.0)[D]	-2.3 (-18.6; 5.1)	–[C]	-2.3 (-12.3; 2.8)[E]	-2.3 (-12.3; 2.8)
	Week 12	-8.4 (-35.6; 2.0)[C]	0.9 (-16.0; 41.7)[D]	-22.8[F]	–[C]	-8.1 (-23.8; 2.7)[H]	-8.1 (-23.8; 2.7)[G]
	Week 12 (LOCF)	-2.0 (-26.6; 0.0)	0.9 (-16.0; 41.7)[D]	-12.4 (-23.6; 5.1)	-30.7	-2.0 (-23.9; 0.9)[C]	-2.0 (-23.1; 2.5)
iscorEB-c	Day 17	-7.0 (-23.6; 3.0)	-11.3 (-12.4; -7.8)[D]	-18.4 (-25.2; 7.1)	-2.2	-11.3 (-23.6; -2.1)[C]	-11.7 (-23.9; -0.8)
	Day 35	-16.1 (-23.9; 3.4)	-10.7 (-32.9; 8.7)[D]	-12.9 (-32.6; 4.0)	–[C]	-13.4 (-25.1; 4.3)[E]	-13.4 (-25.3; 4.3)
	Week 12	-30.7 (-48.4; -10.9)[C]	-4.1 (-39.0; 20.0)[D]	4.6[F]	–[C]	-18.2 (-39.8; -1.9)[H]	-18.2 (-39.8; -1.9)[G]
	Week 12 (LOCF)	-24.4 (-42.3; -7.5)	-4.1 (-39.0; 20.0)[D]	-12.9 (-32.6; 2.3)	-2.2	-12.0 (-37.0; -2.2)[C]	-16.6 (-37.5; -1.9)
iscorEB-p	Day 17	-12.0 (-14.8; 4.0)	-8.1 (-21.4; 66.7)[D]	-23.6 (-30.6; -8.8)	-41.7	-12.1 (-22.9; 3.7)[C]	-12,1 (-21.4; 3.7)
	Day 35	0.0 (-12.0; 30.0)	-7.1 (-27.0; 100.0)[D]	-1.9 (-18.9; 29.1)	-50.0	-7.1 (-24.0; 30.0)[C]	-3.6 (-12.0; 30.0)
	Week 12	-8.0 (-20.0; 20.0)	3.6 (-2.7; 83.3)[D]	6.1 (-29.1; 57.9)	0.0	0.0 (-20.0; 33.3)[C]	0.5 (-20.0; 33.3)

[A]Data are medians (IQR). [B]Values are identical for full analysis set (FAS) and the per-protocol set (PP), because the patient that was excluded from the PP had no post-baseline measurement (which is why the patient's values are not contained in the FAS either). [C]Value from 1 patient missing. [D]Value from 1 patient (FAS) missing. [E]Values from 2 patients missing. [F]Values from 3 patients missing. [G]Values from 4 patients missing. [H]Values from 6 patients missing.

Supplemental Table 3. Percent change in itch score from baseline by age cohort and visit[A]

Visit	Cohort 1 (≥18 yr) FAS & PP N = 7	Cohort 2 (≥12 to <18 yr) FAS N = 4	Cohort 2 (≥12 to <18 yr) PP N = 3	Cohort 3 (≥5 to <12 yr) FAS & PP N = 4	Cohort 4 (≥1 to <5 yr) FAS only N = 1	All patients FAS N = 16	All patients PP N = 14
Day 17	-20.0 (-50.0; 0.0)	-19.7 (-29.2; -7.2)	-14.3 (-25.0; 0.0)	-24.3 (-44.3; 2.5)	-20.0	-20.0 (-31.0; -6.3)	-20.0 (-28.6; 0.0)
Day 35	-37.5 (-40.0; 0.0)	-33.3 (-50.0; -14.3)[B]	-33.3 (-50.0; -14.3)	-51.5 (-60.0; 16.1)	0.0	-37.5 (-42.9; 0.0)[B]	-37.5 (-42.9; -14.3)
Week 12	-25.0 (-50.0; 0.0)	-12.5 (-14.3; 0.0)[B]	-12.5 (-14.3; 0.0)	-31.5 (-61.5; 40.0)	20.0	-14.3 (-42.9; 0.0)[B]	-17.2 (-42.9; 0.0)

[A]Data are medians (IQR). [B]Value from 1 patient missing. FAS, full analysis set; PP, per-protocol set.

Supplemental Table 4. Percent change in pain score from baseline by age cohort and visit[A]

Visit	Cohort 1 (≥18 yr) FAS & PP N = 7	Cohort 2 (≥12 to <18 yr) FAS N = 4	Cohort 2 (≥12 to <18 yr) PP N = 3	Cohort 3 (≥5 to <12 yr) FAS & PP N = 4	Cohort 4 (≥1 to <5 yr) FAS only N = 1	All patients FAS N = 16	All patients PP N = 14
Day 17	0.0 (-37.5; 40.0)	-11.1 (-12.5; 0.0)[B]	-11.8 (-12.5; -11.1)[B]	-28.6 (-28.6; 42.9)[B]	-100.0	-11.8 (-28.6; 16.7)[C]	-11.8 (-28.6; 28.4)[C]
Day 35	0.0 (-42.9; 28.6)	-30.55 (-50.0; -11.1)[C]	-30.55 (-50.0; -11.1)[B]	-42.9 (-57.1; 0.0)[B]	-25.0	-25.0 (-42.9; 0.0)[D]	-24.3 (-46.5; 8.4)[C]
Week 12	-42.9 (-60.0; 16.7)	0.7 (-11.1; 12.5)[C]	0.7 (-11.1; 12.5)[B]	14.3 (-42.9; 28.6)[B]	50.0	-11.1 (-42.9; 16.7)[D]	-24.3 (-43.7; 15.5)[C]

[A]Data are medians (IQR). [B]Value from 1 patient missing. [C]Values from 2 patients missing. [D]Values from 3 patients missing. FAS, full analysis set; PP, per-protocol set.

Supplemental Table 5. Percent change in QOLEB score from baseline by age cohort and visit[A]

Visit	Cohort 1 (≥18 yr) FAS & PP N = 7	Cohort 2 (≥12 to <18 yr) FAS N = 4	Cohort 2 (≥12 to <18 yr) PP N = 3	Cohort 3 (≥5 to <12 yr) FAS & PP N = 4	Cohort 4 (≥1 to <5 yr) FAS only N = 1	All patients FAS N = 16	All patients PP N = 14
Day 17	-3.2 (-15.4; 3.1)	2.1 (-3.2; 10.5)	0.0 (-6.3; 16.7)	0.0 (-8.1; 1.4)	-40.0	0.0 (-13.5; 3.0)	0.0 (-11.5; 2.8)
Day 35	-6.5 (-30.8; -3.4)	0.0 (-8.3; 3.8)[B]	0.0 (-8.3; 3.8)	7.8 (-8.5; 14.6)	440.0	-3.4 (-9.4; 10.0)[B]	-3.6 (-9.4; 5.6)
Week 12	-7.7 (-37.5; 6.9)	9.4 (-8.3; 34.6)[B]	9.4 (-8.3; 34.6)	6.4 (-8.3; 12.7)	520.0	6.5 (-18.8; 15.4)[B]	4.7 (-18.8; 10.0)

[A]Data are medians (IQR). [B]Value from 1 patient missing. FAS, full analysis set; PP, per-protocol set.

Supplemental Table 6. Vital signs[A,B]

Parameter	Baseline (Day 0)	Change from baseline		
		Day 17	Day 35	Week 12
Body temperature, °C		-0.3 (-0.4; 0.2)	0.3 (-0.3; 0.4)[C]	0.2 (-0.4; 0.6)[C]
Blood pressure				
Systolic, mmHg		-2.0 (-6.0; 4.5)	-2.5 (-4.0; 6.0)[C]	2.0 (-4.0; 8.0)[D]
Diastolic, mmHg		5.0 (-3.0; 9.5)	5.5 (2.0; 16.0)[C]	3.0 (-4.0; 8.0)[D]
Heart rate, bpm		2.5 (-2.5; 13.0)	1.5 (-1.0; 6.0)[C]	2.0 (-5.0; 12.0)[D]
Body weight, kg		0.2 (-0.2; 0.6)	0.4 (-0.2; 1.0)[C]	0.1 (-0.5; 1.6)[C]
BMI, kg/m^2		0.1 (-0.1; 0.3)	0.2 (-0.1; 0.6)[C]	0.1 (-0.3; 0.7)[C]

[A]Safety analysis set (N=16). [B]Data are medians (IQR). [C]Values from 2 patients missing.

[D]Values from 3 patients missing.

Supplemental Table 7. Changes in physical examination findings from baseline[A]

Cohort	Patient[B]	Organ system	Visit at	Specification
Cohort 1	1	Extremities	Day 35, Wk 12	Contractures of hands, mutilation of feet (already existing at day 0)
			Day 35, Wk 12	Pain in hands increased[C]
		Lymph nodes	Wk 12	Left inguinal increased[D]
	2	Ears	Day 35, Wk 12	Normal: no lesions present
	3	Lymph nodes	Day 17, Day 35, Wk 12	Normal: no enlarged lymph nodes palpable
		Cardiovascular system	Day 35, Wk 12	Tachycardia (not clinically significant, explainable by history of anemia)
		Ears	Wk 12	Scaly and reddened[E]
		Eyes	Wk 12	Lids reddened, increased secretion (RDEB-related)
		Nose, mouth, throat	Week 12	Cough[F]
	4	Lymph nodes	Day 17, Day 35, Wk 12	Enlarged lymph node in the right posterior neck and the right inguinal area[G]
		Extremities	Day 35, Week 12	Left toe nail[H] surgery and recovered
		Gastrointestinal tract	Day 35, Wk 12	Dysphagia improved, planned esophageal dilation cancelled
		Skin	Wk 12	Skin improved
	5	Skin	Day 35, Wk 12	Wounds on torso and limbs healing
		Lungs and thorax	Wk 12	Few inspiratory crepitations[I]
Cohort 2	6	Eyes	Wk 12	Blister at left eye[J]
	7	Nose, mouth, throat	Day 17	Number of blisters/erosions on oral mucosa increased[K]
			Day 35	New blisters on upper lip[L]
		Skin	Day 17	Wound on the back infected,[M] rest of the skin improved
			Day 35	Wound condition improved, fewer active wounds, no signs of infection
			Wk 12	New wounds on lower legs, non-blanchable, palpable purpuric patch and plaques on feet, legs, thighs, hands, arms[N]
	8	Skin	Day 17	Wounds improved
			Day 35	Wounds improved, new small wound behind left knee[O]
			Wk 12	New blister on left arm[P]

Cohort	Patient[B]	Organ system	Visit at	Specification
	9	Nose, mouth, throat	Day 17	Wound on lip healed
Cohort 3	10	Nose, mouth, throat	Day 17	Erosions on lip improved
			Day 35	Small erosion on palate improved
		Skin	Day 17	Some wounds shrunk
			Day 35	Many wounds shrunk, overall improved
		Ears	Day 35	Less crusts and redness of pinna
			Wk 12	Crusts on both ears greatly improved
		Extremities	Day 35	Fewer wounds, all shrunk and less reddened
			Wk 12	Wound improved

[A]Safety analysis set (N=16). [B]Patients presenting with changes in physical examination findings, numbered consecutively. [C]Documented as TEAE ("Pain in extremity") not related to study treatment. [D]Documented as TEAE ("Lymphadenopathy") related to study treatment. [E]Documented as TEAE ("Ear infection fungal") not related to study treatment. [F]Documented as TEAE ("Cough") not related to study treatment. [G]Documented as TEAE ("Lymphadenopathy") not related to study treatment. [H]Documented as TEAE ("Pseudosyndactyly") not related to study treatment. [I]Documented as TEAE ("Upper respiratory tract infection") not related to study treatment. [J]Documented as TEAE ("Conjunctival bleb") not related to study treatment. [K]Documented as TEAEs ("Oral mucosal blistering" and "Oral mucosa erosion") not related to study treatment. [L]Documented as TEAE ("Lip blister") not related to study treatment. [M]Documented as TEAE ("Wound infection") not related to study treatment. [N]Documented as TEAEs ("Blister" and "Henoch-Schönlein purpura") not related to study treatment. [O]Documented as TEAE ("Skin erosion") not related to study treatment. [P]Documented as TEAE ("Blister") not related to study treatment. TEAE, treatment-emergent adverse event.

Supplemental Table 8. ABCB5^{+} MSC product release data

Date of production	Patient	Lot	Vitality[a)]	Viability[b,c)]	Potency[c)]				
					Immunomodulation		Angiogenesis	Endothelial trans-differentiation	
					IL-1RA secretion[d)]		VEGF secretion[e)]	Tube formation[f)]	
dd/mm/yyyy	#	#	% of cells	% of cells	pg/ml	$Ratio_{stim/unstim}$	pg/ml	Well 1	Well 2
11/02/2019	41-001	C-AL05000-21	96.5	98.2	4,938	2.1	1,117	B/C	C
25.02.2019	41-001	C-AL08000-01	95.5	98.1	52,520	4.8	502	B	A
18/03/2019	41-001	C-AL08000-02	97.0	99.4	1,520	2.0	718	C	E
15/04/2019	21-002	C-AL07000-01	96.5	99.6	4,450	2.5	1,155	B	C
30/04/2019	21-002	C-AL07000-02	95.0	99.6	6,970	3.1	1,284	B	C
20/05/2019	21-002	C-AL07000-03	97.5	99.7	4,910	2.8	1,095	B/C	C
03/06/2019	41-002	C-AL08000-07	98.0	99.6	5,520	2.6	1,045	B	B
18/06/2019	41-002	C-AL05000-29	97.0	99.3	16,180	6.0	412	B	B
08/07/2019	41-002	C-AL08000-09	96.5	99.7	7,450	2.5	1,234	B	C
16/07/2019	21-003	C-AL07000-04	97.5	99.8	5,370	3.0	1,034	C	C
26/07/2019	11-001	C-AL08000-11	96.0	99.4	4,200	2.5	873	B/C	B
30/07/2019	21-003	C-AL10000-01	96.0	99.5	12,730	3.5	1,099	B	C
08/08/2019	42-001	C-AL08000-12	97.0	99.7	7,450	2.5	1,234	B	C
09/08/2019	11-001	C-AL07000-05	95.5	99.5	7,340	3.3	1,174	B	B
12/08/2019	31-001	C-AL10000-02	95.5	99.5	12,730	3.5	1,099	B	C

Date of production	Patient	Lot	Vitality[a)]	Viability[b,c)]	Potency[c)]				
					Immunomodulation		Angiogenesis	Endothelial trans-differentiation	
					IL-1RA secretion[d)]		VEGF secretion[e)]	Tube formation[f)]	
dd/mm/yyyy	#	#	% of cells	% of cells	pg/ml	$Ratio_{stim/unstim}$	pg/ml	Well 1	Well 2
19/08/2019	31-002	C-AL10000-03	97.0	99.6	9,925	3.0	1,015	B/C	C
20/08/2019	21-003	C-AL11000-01	97.0	99.2	4,980	1.4	269	C	C
22/08/2019	42-001	C-AL07000-07	94.5	99.3	7,485	2.8	1,184	B	B/C
26/08/2019	31-001	C-AL11000-02	98.0	99.2	2,770	1.9	464	C	C
30/08/2019	11-001	C-AL11000-03	96.0	99.2	4,980	1.4	269	C	C
30/08/2019	11-002	C-AL08000-13	97.5	99.7	7,450	2.5	1,234	B	C
02/09/2019	31-002	C-AL11000-04	93.5	98.3	5,250	1.4	287	B	C
12/09/2019	42-001	C-AL11000-05	87.5	98.3	5,250	1.4	287	B	C
16/09/2019	31-001	C-AL10000-04	96.5	99.7	12,080	2.3	1,086	B	B
18/09/2019	11-002	C-AL07000-13	95.0	99.4	7,820	3.4	853	B	B
23/09/2019	31-002	C-AL11000-06	91.7	99.3	8,330	2.9	603	C	C
27/09/2019	42-002	C-AL07000-16	97.0	99.7	8,300	3.6	911	B	C
02/10/2019	11-002	C-AL11000-07	92.5	98.3	5,250	1.4	287	B	C
11/10/2019	42-002	C-AL10000-05	96.5	99.6	13,840	3.4	1,233	A	D
28/10/2019	42-003	C-AL07000-17	95.0	99.4	7,035	2.9	933	B/C	B
01/11/2019	42-002	C-AL11000-08	93.5	99.6	2,149	2.6	364	B	B
06/11/2019	11-003	C-AL07000-18	97.0	99.2	7,960	2.3	1,589	C	C

Date of production	Patient	Lot	Vitality[a)]	Viability[b,c)]	Potency[c)]					
					Immunomodulation		Angiogenesis	Endothelial trans-differentiation	
					IL-1RA secretion[d)]		VEGF secretion[e)]	Tube formation[f)]	
dd/mm/yyyy	#	#	% of cells	% of cells	pg/ml	$Ratio_{stim/unstim}$	pg/ml	Well 1	Well 2
14/11/2019	42-003	C-AL11000-10	93.5	99.6	2,149	2.6	364	B	B
21/11/2019	11-003	C-AL10000-08	97.5	99.6	13,840	3.4	1,233	A	D
02/12/2019	42-004	C-AL11000-11	95.0	99.6	2,149	2.6	364	B	B
11/12/2019	11-003	C-AL11000-12	94.1	99.7	2,096	2.2	425	C	C
19/12/2019	42-004	C-AL08000-15	97.2	99.4	13,120	4.2	1,378	A	C
09/01/2020	42-004	C-AL10000-09	97.1	99.7	8,680	2,2	1,086	B	C
15/01/2020	51-001	C-AL11000-14	97.4	99.2	2,770	1.9	464	C	C
03/02/2020	51-001	C-AL10000-10	97.4	99.5	12,730	3.5	1,099	B	C
03/02/2020	51-002	C-AL11000-15	94.7	98.3	5,250	1.4	287	B	C
14/02/2020	11-004	C-AL08000-17	96.1	99.6	10,285	3.4	1,306	A/A	C
17/02/2020	51-001	C-AL11000-16	94.1	99.3	8,330	2.9	603	C	C
17/02/2020	51-002	C-AL10000-11	92.5	99.4	10,740	2.0	350	B	C
28/02/2020	11-004	C-AL07000-33	95.5	99.2	7,960	2.3	1,589	C	C
09/03/2020	51-002	C-AL11000-17	94.2	99.3	8,330	2.9	609	C	C
**Specification**			**≥ 75%**	**≥ 90%**	**> 125**	**> 1**	> 46.9	**A, B or C**	
**% of products fulfilling the specification**			**100%**	**100%**	**100%**	**100%**	**100%**	**100%**	

For more detailed description of the release testing algorithm and the methods see Ballikaya S, et al. 2020[g)].

[a]Defined as percentage of live cells determined as propidium iodide-excluding cells.

[b]Defined as percentage of metabolically active cells determined as cells converting calcein acetoxymethylester to calcein.

[c]Values were adopted from drug substance release testing; transferability of the results from drug substance release testing onto the final drug product was demonstrated in stability studies and method validation (Ballikaya et al., 2020[g])).

[d]Determined after cocultivation with M1-polarized macrophages to evaluate the immunomodulatory potency of the $ABCB5^+$ MSCs.

[e]Determined after 48 h culture under hypoxic conditions to evaluate the angiogenic potency of the $ABCB5^+$ MSCs.

[f]Determined after 19-22 h incubation on extracellular matrix gel to evaluate the endothelial trans-differentiation capacity of the $ABCB5^+$ MSCs. A, tubular branches of several cells forming a defined network-like structure; B, tubular branches of several cells clustering together forming broad strands, formation of syncytia, areas of high cellular density lacking formation of tubular branches; C, cells clustering together, building nodes and forming tubular branches that connect the nodes with each other; D, only sporadic cells form tubular branches, partial node formation, but no or nearly no connections between nodes, no or only sporadic apoptotic cells; E, largely apoptotic cells, no or only sporadic tubular branches; F, no tubular branches.

[g]Ballikaya S, et al. Process data of allogeneic ex vivo-expanded $ABCB5^+$ mesenchymal stromal cells for human use: off-the-shelf GMP-manufactured donor-independent ATMP. Stem Cell Res Ther. 2020;11(1):482.
