# Supplementary material for: Clinical trial of ABCB5+ mesenchymal stem cells for recessive dystrophic epidermolysis bullosa
Source: JCI Insight. 2021 Nov 22;6(22):e151922. doi: 10.1172/jci.insight.151922 (PMC8663784; doi:10.1172/jci.insight.151922)
Supplement: ICMJE disclosure forms [file jciinsight-6-151922-s056.pdf]

## ICMJE DISCLOSURE FORM

**Date: 22.07.2021**________________________________________________________________________
**Your Name: Dimitra Kiritsi**__________________________________________________________________
**Manuscript Title: Phase I/IIa clinical trial of ABCB5-positive dermal mesenchymal stem cells for recessive dystrophic epidermolysis bullosa**_______________________________________________________________
**Manuscript number (if known):**______________________________________________________________

**In the interest of transparency, we ask you to disclose all relationships/activities/interests listed below that are related to the content of your manuscript. "Related" means any relation with for-profit or not-for-profit third parties whose interests may be affected by the content of the manuscript. Disclosure represents a commitment to transparency and does not necessarily indicate a bias. If you are in doubt about whether to list a relationship/activity/interest, it is preferable that you do so.**

**The following questions apply to the author's relationships/activities/interests as they relate to the current manuscript only.**

**The author's relationships/activities/interests should be defined broadly. For example, if your manuscript pertains to the epidemiology of hypertension, you should declare all relationships with manufacturers of antihypertensive medication, even if that medication is not mentioned in the manuscript.**

**In item #1 below, report all support for the work reported in this manuscript without time limit. For all other items, the time frame for disclosure is the past 36 months.**

| | | **Name all entities with whom you have this relationship or indicate none (add rows as needed)** | **Specifications/Comments (e.g., if payments were made to you or to your institution)** |
|---|---|---|---|
| | | **Time frame: Since the initial planning of the work** | |
| 1 | All support for the present manuscript (e.g., funding, provision of study materials, medical writing, article processing charges, etc.) **No time limit for this item.** | DK's work is funded by the Berta-Ottenstein-Programme for Advanced Clinician Scientists, Faculty of Medicine, University of Freiburg, by the German Research Foundation (DFG) through SFB1160 project B03, SFB-1479 – Project ID: 441891347 and KI1795/2-1 and the Fritz Thyssen Foundation. All funding organizations provided funding covering the conduct of the study and the preparation of the manuscript, but were not | Payments made to the institution. |

| | | | |
|---|---|---|---|
| | | involved in the design of the study. | |
| | | | |
| | | | |
| | | | |
| | | | |
| | | | |
| | | | |
| | | | |
| **Time frame: past 36 months** | | | |
| 2 | Grants or contracts from any entity (if not indicated in item #1 above). | | |
| | | Has received funding from Rheacell GmbH for the conduct of scientific studies with ABCB5+ cells in a mouse model with DEB. | Payments made to the institution. |
| | | | |
| 3 | Royalties or licenses | ____ None | |
| | | | |
| | | | |
| 4 | Consulting fees | ____ None | |
| | | | |
| | | | |
| 5 | Payment or honoraria for lectures, presentations, speakers bureaus, manuscript writing or educational events | Has received honoraria from Rheacell GmbH for consulting on the design of the trial protocol. | Payments to DK. |
| | | | |
| | | Honoraria for consulting of Amryt Pharmaceuticals, FIBRX Derm Inc, Colzyk AB. | |
| 6 | Payment for expert testimony | ____ None | |
| | | | |
| | | | |
| 7 | Support for attending meetings and/or travel | ____ None | |
| | | | |
| | | | |
| 8 | Patents planned, issued or pending | ____ None | |
| | | | |
| | | | |
| 9 | Participation on a Data Safety Monitoring Board or Advisory Board | Is participating in the Data Safety Monitoring Board of the trial, as Principal Investigator of the Trial. | |

| | | | |
|---|---|---|---|
| | | | |
| | | | |
| | | | |
| 10 | Leadership or fiduciary role in other board, society, committee or advocacy group, paid or unpaid | ____ None | |
| | | | |
| | | | |
| 11 | Stock or stock options | ____ None | |
| | | | |
| | | | |
| 12 | Receipt of equipment, materials, drugs, medical writing, gifts or other services | ____ None | |
| | | | |
| | | | |
| 13 | Other financial or non-financial interests | ____ None | |
| | | | |
| | | | |

**Please place an "X" next to the following statement to indicate your agreement:**

**x___ I certify that I have answered every question and have not altered the wording of any of the questions on this form.**

## ICMJE DISCLOSURE FORM

**Date:** 27.07.2021
**Your Name:** Kathrin Dieter
**Manuscript Title:** Phase I/IIa clinical trial of ABCB5-positive dermal mesenchymal stem cells for recessive dystrophic epidermolysis bullosa
**Manuscript number (if known):** 151922-INS-CMED-RV-3

**In the interest of transparency, we ask you to disclose all relationships/activities/interests listed below that are related to the content of your manuscript. "Related" means any relation with for-profit or not-for-profit third parties whose interests may be affected by the content of the manuscript. Disclosure represents a commitment to transparency and does not necessarily indicate a bias. If you are in doubt about whether to list a relationship/activity/interest, it is preferable that you do so.**

**The following questions apply to the author's relationships/activities/interests as they relate to the current manuscript only.**

**The author's relationships/activities/interests should be defined broadly. For example, if your manuscript pertains to the epidemiology of hypertension, you should declare all relationships with manufacturers of antihypertensive medication, even if that medication is not mentioned in the manuscript.**

**In item #1 below, report all support for the work reported in this manuscript without time limit. For all other items, the time frame for disclosure is the past 36 months.**

| | | Name all entities with whom you have this relationship or indicate none (add rows as needed) | Specifications/Comments (e.g., if payments were made to you or to your institution) |
|---|---|---|---|
| | | **Time frame: Since the initial planning of the work** | |
| 1 | All support for the present manuscript (e.g., funding, provision of study materials, medical writing, article processing charges, etc.) **No time limit for this item.** | X None | |
| | | | |
| | | | |
| | | | |
| | | | |
| | | | |
| | | | |
| | | | |
| | | **Time frame: past 36 months** | |
| 2 | Grants or contracts from any entity (if not indicated in item #1 above). | X None | |
| | | | |
| | | | |
| 3 | Royalties or licenses | X None | |
| | | | |

| | | | |
|---|---|---|---|
| 4 | Consulting fees | x None | |
| | | | |
| | | | |
| 5 | Payment or honoraria for lectures, presentations, speakers bureaus, manuscript writing or educational events | X None | |
| | | | |
| | | | |
| 6 | Payment for expert testimony | X None | |
| | | | |
| | | | |
| 7 | Support for attending meetings and/or travel | X None | |
| | | | |
| | | | |
| 8 | Patents planned, issued or pending | X None | |
| | | | |
| | | | |
| 9 | Participation on a Data Safety Monitoring Board or Advisory Board | X None | |
| | | | |
| | | | |
| 10 | Leadership or fiduciary role in other board, society, committee or advocacy group, paid or unpaid | X None | |
| | | | |
| | | | |
| 11 | Stock or stock options | X None | |
| | | | |
| | | | |
| 12 | Receipt of equipment, materials, drugs, medical writing, gifts or other services | X None | |
| | | | |
| | | | |
| 13 | Other financial or non-financial interests | RHEACELL GmbH & Co.KG | Employee |
| | | | |
| | | | |

**Please place an "X" next to the following statement to indicate your agreement:**

**X I certify that I have answered every question and have not altered the wording of any of the questions on this form.**

## ICMJE DISCLOSURE FORM

**Date:** **27.07.2021**
**Your Name:** **Elke Niebergall-Roth**
**Manuscript Title:** **Phase I/IIa clinical trial of ABCB5-positive dermal mesenchymal stem cells for recessive dystrophic epidermolysis bullosa**
**Manuscript number (if known):** **151922-INS-CMED-RV-3**

**In the interest of transparency, we ask you to disclose all relationships/activities/interests listed below that are related to the content of your manuscript. "Related" means any relation with for-profit or not-for-profit third parties whose interests may be affected by the content of the manuscript. Disclosure represents a commitment to transparency and does not necessarily indicate a bias. If you are in doubt about whether to list a relationship/activity/interest, it is preferable that you do so.**

**The following questions apply to the author's relationships/activities/interests as they relate to the current manuscript only.**

**The author's relationships/activities/interests should be defined broadly. For example, if your manuscript pertains to the epidemiology of hypertension, you should declare all relationships with manufacturers of antihypertensive medication, even if that medication is not mentioned in the manuscript.**

**In item #1 below, report all support for the work reported in this manuscript without time limit. For all other items, the time frame for disclosure is the past 36 months.**

| | | **Name all entities with whom you have this relationship or indicate none (add rows as needed)** | **Specifications/Comments (e.g., if payments were made to you or to your institution)** |
|---|---|---|---|
| | | **Time frame: Since the initial planning of the work** | |
| 1 | All support for the present manuscript (e.g., funding, provision of study materials, medical writing, article processing charges, etc.) **No time limit for this item.** | X None | |
| | | | |
| | | | |
| | | | |
| | | | |
| | | | |
| | | | |
| | | | |
| | | **Time frame: past 36 months** | |
| 2 | Grants or contracts from any entity (if not indicated in item #1 above). | X None | |
| | | | |
| | | | |
| 3 | Royalties or licenses | X None | |
| | | | |

| | | | |
|---|---|---|---|
| 4 | Consulting fees | x None | |
| | | | |
| | | | |
| 5 | Payment or honoraria for lectures, presentations, speakers bureaus, manuscript writing or educational events | X None | |
| | | | |
| | | | |
| 6 | Payment for expert testimony | X None | |
| | | | |
| | | | |
| 7 | Support for attending meetings and/or travel | X None | |
| | | | |
| | | | |
| 8 | Patents planned, issued or pending | X None | |
| | | | |
| | | | |
| 9 | Participation on a Data Safety Monitoring Board or Advisory Board | X None | |
| | | | |
| | | | |
| 10 | Leadership or fiduciary role in other board, society, committee or advocacy group, paid or unpaid | X None | |
| | | | |
| | | | |
| 11 | Stock or stock options | X None | |
| | | | |
| | | | |
| 12 | Receipt of equipment, materials, drugs, medical writing, gifts or other services | X None | |
| | | | |
| | | | |
| 13 | Other financial or non-financial interests | TICEBA GmbH | Employee |
| | | | |
| | | | |

**Please place an "X" next to the following statement to indicate your agreement:**

**X I certify that I have answered every question and have not altered the wording of any of the questions on this form.**

**ICMJE DISCLOSURE FORM**

**Date:** **27.07.2021**
**Your Name:** **Silvia Fluhr**
**Manuscript Title:** **Phase I/IIa clinical trial of ABCB5-positive dermal mesenchymal stem cells for recessive dystrophic epidermolysis bullosa**
**Manuscript number (if known):** **151922-INS-CMED-RV-3**

**In the interest of transparency, we ask you to disclose all relationships/activities/interests listed below that are related to the content of your manuscript. "Related" means any relation with for-profit or not-for-profit third parties whose interests may be affected by the content of the manuscript. Disclosure represents a commitment to transparency and does not necessarily indicate a bias. If you are in doubt about whether to list a relationship/activity/interest, it is preferable that you do so.**

**The following questions apply to the author's relationships/activities/interests as they relate to the current manuscript only.**

**The author's relationships/activities/interests should be defined broadly. For example, if your manuscript pertains to the epidemiology of hypertension, you should declare all relationships with manufacturers of antihypertensive medication, even if that medication is not mentioned in the manuscript.**

**In item #1 below, report all support for the work reported in this manuscript without time limit. For all other items, the time frame for disclosure is the past 36 months.**

| | | **Name all entities with whom you have this relationship or indicate none (add rows as needed)** | **Specifications/Comments (e.g., if payments were made to you or to your institution)** |
|---|---|---|---|
| | | **Time frame: Since the initial planning of the work** | |
| 1 | All support for the present manuscript (e.g., funding, provision of study materials, medical writing, article processing charges, etc.) **No time limit for this item.** | X None | |
| | | | |
| | | | |
| | | | |
| | | | |
| | | | |
| | | | |
| | | | |
| | | **Time frame: past 36 months** | |
| 2 | Grants or contracts from any entity (if not indicated in item #1 above). | X None | |
| | | | |
| | | | |
| 3 | Royalties or licenses | X None | |
| | | | |

| | | | |
|---|---|---|---|
| 4 | Consulting fees | x None | |
| | | | |
| | | | |
| 5 | Payment or honoraria for lectures, presentations, speakers bureaus, manuscript writing or educational events | X None | |
| | | | |
| | | | |
| 6 | Payment for expert testimony | X None | |
| | | | |
| | | | |
| 7 | Support for attending meetings and/or travel | X None | |
| | | | |
| | | | |
| 8 | Patents planned, issued or pending | X None | |
| | | | |
| | | | |
| 9 | Participation on a Data Safety Monitoring Board or Advisory Board | X None | |
| | | | |
| | | | |
| 10 | Leadership or fiduciary role in other board, society, committee or advocacy group, paid or unpaid | X None | |
| | | | |
| | | | |
| 11 | Stock or stock options | X None | |
| | | | |
| | | | |
| 12 | Receipt of equipment, materials, drugs, medical writing, gifts or other services | X None | |
| | | | |
| | | | |
| 13 | Other financial or non-financial interests | RHEACELL GmbH & Co.KG | Employee |
| | | | |
| | | | |

**Please place an "X" next to the following statement to indicate your agreement:**

**X I certify that I have answered every question and have not altered the wording of any of the questions on this form.**

## ICMJE DISCLOSURE FORM

**Date:** 27.07.2021
**Your Name:** Cristina Daniele
**Manuscript Title:** Phase I/IIa clinical trial of ABCB5-positive dermal mesenchymal stem cells for recessive dystrophic epidermolysis bullosa
**Manuscript number (if known):** 151922-INS-CMED-RV-3

**In the interest of transparency, we ask you to disclose all relationships/activities/interests listed below that are related to the content of your manuscript. "Related" means any relation with for-profit or not-for-profit third parties whose interests may be affected by the content of the manuscript. Disclosure represents a commitment to transparency and does not necessarily indicate a bias. If you are in doubt about whether to list a relationship/activity/interest, it is preferable that you do so.**

**The following questions apply to the author's relationships/activities/interests as they relate to the <u>current manuscript only</u>.**

**The author's relationships/activities/interests should be <u>defined broadly</u>. For example, if your manuscript pertains to the epidemiology of hypertension, you should declare all relationships with manufacturers of antihypertensive medication, even if that medication is not mentioned in the manuscript.**

**In item #1 below, report all support for the work reported in this manuscript without time limit. For all other items, the time frame for disclosure is the past 36 months.**

| | | **Name all entities with whom you have this relationship or indicate none (add rows as needed)** | **Specifications/Comments (e.g., if payments were made to you or to your institution)** |
|---|---|---|---|
| **Time frame: Since the initial planning of the work** | | | |
| 1 | All support for the present manuscript (e.g., funding, provision of study materials, medical writing, article processing charges, etc.) **No time limit for this item.** | X None | |
| | | | |
| | | | |
| | | | |
| | | | |
| | | | |
| | | | |
| | | | |
| **Time frame: past 36 months** | | | |
| 2 | Grants or contracts from any entity (if not indicated in item #1 above). | X None | |
| | | | |
| | | | |
| 3 | Royalties or licenses | X None | |
| | | | |

| | | | |
|---|---|---|---|
| 4 | Consulting fees | x None | |
| | | | |
| | | | |
| 5 | Payment or honoraria for lectures, presentations, speakers bureaus, manuscript writing or educational events | X None | |
| | | | |
| | | | |
| 6 | Payment for expert testimony | X None | |
| | | | |
| | | | |
| 7 | Support for attending meetings and/or travel | X None | |
| | | | |
| | | | |
| 8 | Patents planned, issued or pending | X None | |
| | | | |
| | | | |
| 9 | Participation on a Data Safety Monitoring Board or Advisory Board | X None | |
| | | | |
| | | | |
| 10 | Leadership or fiduciary role in other board, society, committee or advocacy group, paid or unpaid | X None | |
| | | | |
| | | | |
| 11 | Stock or stock options | X None | |
| | | | |
| | | | |
| 12 | Receipt of equipment, materials, drugs, medical writing, gifts or other services | X None | |
| | | | |
| | | | |
| 13 | Other financial or non-financial interests | RHEACELL GmbH & Co.KG | Employee |
| | | | |
| | | | |

**Please place an "X" next to the following statement to indicate your agreement:**

**X I certify that I have answered every question and have not altered the wording of any of the questions on this form.**

# ICMJE DISCLOSURE FORM

**Date:** 27.07.2021
**Your Name:** Jasmina Esterlechner
**Manuscript Title:** Phase I/IIa clinical trial of ABCB5-positive dermal mesenchymal stem cells for recessive dystrophic epidermolysis bullosa
**Manuscript number (if known):** 151922-INS-CMED-RV-3

**In the interest of transparency, we ask you to disclose all relationships/activities/interests listed below that are related to the content of your manuscript. "Related" means any relation with for-profit or not-for-profit third parties whose interests may be affected by the content of the manuscript. Disclosure represents a commitment to transparency and does not necessarily indicate a bias. If you are in doubt about whether to list a relationship/activity/interest, it is preferable that you do so.**

**The following questions apply to the author's relationships/activities/interests as they relate to the current manuscript only.**

**The author's relationships/activities/interests should be defined broadly. For example, if your manuscript pertains to the epidemiology of hypertension, you should declare all relationships with manufacturers of antihypertensive medication, even if that medication is not mentioned in the manuscript.**

**In item #1 below, report all support for the work reported in this manuscript without time limit. For all other items, the time frame for disclosure is the past 36 months.**

| | | Name all entities with whom you have this relationship or indicate none (add rows as needed) | Specifications/Comments (e.g., if payments were made to you or to your institution) |
|---|---|---|---|
| | | **Time frame: Since the initial planning of the work** | |
| 1 | All support for the present manuscript (e.g., funding, provision of study materials, medical writing, article processing charges, etc.) **No time limit for this item.** | X None | |
| | | | |
| | | | |
| | | | |
| | | | |
| | | | |
| | | | |
| | | | |
| | | **Time frame: past 36 months** | |
| 2 | Grants or contracts from any entity (if not indicated in item #1 above). | X None | |
| | | | |
| | | | |
| 3 | Royalties or licenses | X None | |
| | | | |

| | | | |
|---|---|---|---|
| 4 | Consulting fees | x None | |
| | | | |
| | | | |
| 5 | Payment or honoraria for lectures, presentations, speakers bureaus, manuscript writing or educational events | X None | |
| | | | |
| | | | |
| 6 | Payment for expert testimony | X None | |
| | | | |
| | | | |
| 7 | Support for attending meetings and/or travel | X None | |
| | | | |
| | | | |
| 8 | Patents planned, issued or pending | X None | |
| | | | |
| | | | |
| 9 | Participation on a Data Safety Monitoring Board or Advisory Board | X None | |
| | | | |
| | | | |
| 10 | Leadership or fiduciary role in other board, society, committee or advocacy group, paid or unpaid | X None | |
| | | | |
| | | | |
| 11 | Stock or stock options | X None | |
| | | | |
| | | | |
| 12 | Receipt of equipment, materials, drugs, medical writing, gifts or other services | X None | |
| | | | |
| | | | |
| 13 | Other financial or non-financial interests | TICEBA GmbH | Employee |
| | | | |
| | | | |

**Please place an "X" next to the following statement to indicate your agreement:**

**X I certify that I have answered every question and have not altered the wording of any of the questions on this form.**

## ICMJE DISCLOSURE FORM

**Date:** 27.07.2021
**Your Name:** Samar Sadeghi
**Manuscript Title:** Phase I/IIa clinical trial of ABCB5-positive dermal mesenchymal stem cells for recessive dystrophic epidermolysis bullosa
**Manuscript number (if known):** 151922-INS-CMED-RV-3

**In the interest of transparency, we ask you to disclose all relationships/activities/interests listed below that are related to the content of your manuscript. "Related" means any relation with for-profit or not-for-profit third parties whose interests may be affected by the content of the manuscript. Disclosure represents a commitment to transparency and does not necessarily indicate a bias. If you are in doubt about whether to list a relationship/activity/interest, it is preferable that you do so.**

**The following questions apply to the author's relationships/activities/interests as they relate to the current manuscript only.**

**The author's relationships/activities/interests should be defined broadly. For example, if your manuscript pertains to the epidemiology of hypertension, you should declare all relationships with manufacturers of antihypertensive medication, even if that medication is not mentioned in the manuscript.**

**In item #1 below, report all support for the work reported in this manuscript without time limit. For all other items, the time frame for disclosure is the past 36 months.**

| | | **Name all entities with whom you have this relationship or indicate none (add rows as needed)** | **Specifications/Comments (e.g., if payments were made to you or to your institution)** |
|---|---|---|---|
| **Time frame: Since the initial planning of the work** | | | |
| 1 | All support for the present manuscript (e.g., funding, provision of study materials, medical writing, article processing charges, etc.) **No time limit for this item.** | X None | |
| | | | |
| | | | |
| | | | |
| | | | |
| | | | |
| | | | |
| | | | |
| **Time frame: past 36 months** | | | |
| 2 | Grants or contracts from any entity (if not indicated in item #1 above). | X None | |
| | | | |
| | | | |
| 3 | Royalties or licenses | X None | |
| | | | |

| | | | |
|---|---|---|---|
| 4 | Consulting fees | x None | |
| | | | |
| | | | |
| 5 | Payment or honoraria for lectures, presentations, speakers bureaus, manuscript writing or educational events | X None | |
| | | | |
| | | | |
| 6 | Payment for expert testimony | X None | |
| | | | |
| | | | |
| 7 | Support for attending meetings and/or travel | X None | |
| | | | |
| | | | |
| 8 | Patents planned, issued or pending | X None | |
| | | | |
| | | | |
| 9 | Participation on a Data Safety Monitoring Board or Advisory Board | X None | |
| | | | |
| | | | |
| 10 | Leadership or fiduciary role in other board, society, committee or advocacy group, paid or unpaid | X None | |
| | | | |
| | | | |
| 11 | Stock or stock options | X None | |
| | | | |
| | | | |
| 12 | Receipt of equipment, materials, drugs, medical writing, gifts or other services | X None | |
| | | | |
| | | | |
| 13 | Other financial or non-financial interests | TICEBA GmbH | Employee |
| | | | |
| | | | |

**Please place an "X" next to the following statement to indicate your agreement:**

**X I certify that I have answered every question and have not altered the wording of any of the questions on this form.**

# ICMJE DISCLOSURE FORM

**Date:** 27.07.2021
**Your Name:** Seda Ballikaya
**Manuscript Title:** Phase I/IIa clinical trial of ABCB5-positive dermal mesenchymal stem cells for recessive dystrophic epidermolysis bullosa
**Manuscript number (if known):** 151922-INS-CMED-RV-3

**In the interest of transparency, we ask you to disclose all relationships/activities/interests listed below that are related to the content of your manuscript. "Related" means any relation with for-profit or not-for-profit third parties whose interests may be affected by the content of the manuscript. Disclosure represents a commitment to transparency and does not necessarily indicate a bias. If you are in doubt about whether to list a relationship/activity/interest, it is preferable that you do so.**

**The following questions apply to the author's relationships/activities/interests as they relate to the current manuscript only.**

**The author's relationships/activities/interests should be defined broadly. For example, if your manuscript pertains to the epidemiology of hypertension, you should declare all relationships with manufacturers of antihypertensive medication, even if that medication is not mentioned in the manuscript.**

**In item #1 below, report all support for the work reported in this manuscript without time limit. For all other items, the time frame for disclosure is the past 36 months.**

| | | **Name all entities with whom you have this relationship or indicate none (add rows as needed)** | **Specifications/Comments (e.g., if payments were made to you or to your institution)** |
|---|---|---|---|
| | | **Time frame: Since the initial planning of the work** | |
| 1 | All support for the present manuscript (e.g., funding, provision of study materials, medical writing, article processing charges, etc.) **No time limit for this item.** | X None | |
| | | | |
| | | | |
| | | | |
| | | | |
| | | | |
| | | | |
| | | | |
| | | **Time frame: past 36 months** | |
| 2 | Grants or contracts from any entity (if not indicated in item #1 above). | X None | |
| | | | |
| | | | |
| 3 | Royalties or licenses | X None | |
| | | | |

| | | | |
|---|---|---|---|
| 4 | Consulting fees | x None | |
| | | | |
| | | | |
| 5 | Payment or honoraria for lectures, presentations, speakers bureaus, manuscript writing or educational events | X None | |
| | | | |
| | | | |
| 6 | Payment for expert testimony | X None | |
| | | | |
| | | | |
| 7 | Support for attending meetings and/or travel | X None | |
| | | | |
| | | | |
| 8 | Patents planned, issued or pending | X None | |
| | | | |
| | | | |
| 9 | Participation on a Data Safety Monitoring Board or Advisory Board | X None | |
| | | | |
| | | | |
| 10 | Leadership or fiduciary role in other board, society, committee or advocacy group, paid or unpaid | X None | |
| | | | |
| | | | |
| 11 | Stock or stock options | X None | |
| | | | |
| | | | |
| 12 | Receipt of equipment, materials, drugs, medical writing, gifts or other services | X None | |
| | | | |
| | | | |
| 13 | Other financial or non-financial interests | TICEBA GmbH | Employee |
| | | | |
| | | | |

**Please place an "X" next to the following statement to indicate your agreement:**

**X I certify that I have answered every question and have not altered the wording of any of the questions on this form.**

# ICMJE DISCLOSURE FORM

**Date:** 27.07.2021
**Your Name:** Leoni Erdinger
**Manuscript Title:** Phase I/IIa clinical trial of ABCB5-positive dermal mesenchymal stem cells for recessive dystrophic epidermolysis bullosa
**Manuscript number (if known):** 151922-INS-CMED-RV-3

**In the interest of transparency, we ask you to disclose all relationships/activities/interests listed below that are related to the content of your manuscript. "Related" means any relation with for-profit or not-for-profit third parties whose interests may be affected by the content of the manuscript. Disclosure represents a commitment to transparency and does not necessarily indicate a bias. If you are in doubt about whether to list a relationship/activity/interest, it is preferable that you do so.**

**The following questions apply to the author's relationships/activities/interests as they relate to the current manuscript only.**

**The author's relationships/activities/interests should be defined broadly. For example, if your manuscript pertains to the epidemiology of hypertension, you should declare all relationships with manufacturers of antihypertensive medication, even if that medication is not mentioned in the manuscript.**

**In item #1 below, report all support for the work reported in this manuscript without time limit. For all other items, the time frame for disclosure is the past 36 months.**

| | | Name all entities with whom you have this relationship or indicate none (add rows as needed) | Specifications/Comments (e.g., if payments were made to you or to your institution) |
|---|---|---|---|
| | | **Time frame: Since the initial planning of the work** | |
| 1 | All support for the present manuscript (e.g., funding, provision of study materials, medical writing, article processing charges, etc.) **No time limit for this item.** | X None | |
| | | | |
| | | | |
| | | | |
| | | | |
| | | | |
| | | | |
| | | | |
| | | **Time frame: past 36 months** | |
| 2 | Grants or contracts from any entity (if not indicated in item #1 above). | X None | |
| | | | |
| | | | |
| 3 | Royalties or licenses | X None | |
| | | | |

| | | | |
|---|---|---|---|
| 4 | Consulting fees | x None | |
| | | | |
| | | | |
| 5 | Payment or honoraria for lectures, presentations, speakers bureaus, manuscript writing or educational events | X None | |
| | | | |
| | | | |
| 6 | Payment for expert testimony | X None | |
| | | | |
| | | | |
| 7 | Support for attending meetings and/or travel | X None | |
| | | | |
| | | | |
| 8 | Patents planned, issued or pending | X None | |
| | | | |
| | | | |
| 9 | Participation on a Data Safety Monitoring Board or Advisory Board | X None | |
| | | | |
| | | | |
| 10 | Leadership or fiduciary role in other board, society, committee or advocacy group, paid or unpaid | X None | |
| | | | |
| | | | |
| 11 | Stock or stock options | X None | |
| | | | |
| | | | |
| 12 | Receipt of equipment, materials, drugs, medical writing, gifts or other services | X None | |
| | | | |
| | | | |
| 13 | Other financial or non-financial interests | RHEACELL GmbH & Co.KG | Employee |
| | | | |
| | | | |

**Please place an "X" next to the following statement to indicate your agreement:**

**X I certify that I have answered every question and have not altered the wording of any of the questions on this form.**

## ICMJE DISCLOSURE FORM

**Date: 29.07.2021**
**Your Name: Franziska Schauer**
**Manuscript Title: Phase I/IIa clinical trial of ABCB5-positive dermal mesenchymal stem cells for recessive dystrophic epidermolysis bullosa**
**Manuscript number (if known):**

**In the interest of transparency, we ask you to disclose all relationships/activities/interests listed below that are related to the content of your manuscript. "Related" means any relation with for-profit or not-for-profit third parties whose interests may be affected by the content of the manuscript. Disclosure represents a commitment to transparency and does not necessarily indicate a bias. If you are in doubt about whether to list a relationship/activity/interest, it is preferable that you do so.**

**The following questions apply to the author's relationships/activities/interests as they relate to the current manuscript only.**

**The author's relationships/activities/interests should be defined broadly. For example, if your manuscript pertains to the epidemiology of hypertension, you should declare all relationships with manufacturers of antihypertensive medication, even if that medication is not mentioned in the manuscript.**

**In item #1 below, report all support for the work reported in this manuscript without time limit. For all other items, the time frame for disclosure is the past 36 months.**

| | | **Name all entities with whom you have this relationship or indicate none (add rows as needed)** | **Specifications/Comments (e.g., if payments were made to you or to your institution)** |
|---|---|---|---|
| **Time frame: Since the initial planning of the work** | | | |
| 1 | All support for the present manuscript (e.g., funding, provision of study materials, medical writing, article processing charges, etc.) **No time limit for this item.** | ____ None<br>FS is supported by the Berta-Ottenstein Advanced Clinician Scientist Programme of the University of Freiburg | |
| | | | |
| | | | |
| | | | |
| | | | |
| | | | |
| | | | |
| | | | |
| **Time frame: past 36 months** | | | |
| 2 | | __x__ None | |

| | | | |
|---|---|---|---|
| | Grants or contracts from any entity (if not indicated in item #1 above). | | |
| | | | |
| 3 | Royalties or licenses | __x__ None | |
| | | | |
| | | | |
| 4 | Consulting fees | ____ None<br>FS received fees from Amryt Pharma. | |
| | | | |
| | | | |
| 5 | Payment or honoraria for lectures, presentations, speakers bureaus, manuscript writing or educational events | __x__ None | |
| | | | |
| | | | |
| 6 | Payment for expert testimony | __x__ None | |
| | | | |
| | | | |
| 7 | Support for attending meetings and/or travel | _x___ None | |
| | | | |
| | | | |
| 8 | Patents planned, issued or pending | __x__ None | |
| | | | |
| | | | |
| 9 | Participation on a Data Safety Monitoring Board or Advisory Board | __x__ None | |
| | | | |
| | | | |
| 10 | Leadership or fiduciary role in other board, society, committee or advocacy group, paid or unpaid | ___x_ None | |
| | | | |
| | | | |
| 11 | Stock or stock options | __x__ None | |
| | | | |
| | | | |
| 12 | Receipt of equipment, materials, drugs, medical writing, gifts or other services | __x__ None | |
| | | | |
| | | | |
| 13 | Other financial or non-financial interests | __x__ None | |
| | | | |
| | | | |

**Please place an "X" next to the following statement to indicate your agreement:**

**__x_ I certify that I have answered every question and have not altered the wording of any of the questions on this form.**

## ICMJE DISCLOSURE FORM

**Date: 27.07.2021**
**Your Name: Stella Gewert**
**Manuscript Title: Phase I/IIa clinical trial of ABCB5-positive dermal mesenchymal stem cells for recessive dystrophic epidermolysis bullosa**
**Manuscript number (if known):__________________________________________________________**

**In the interest of transparency, we ask you to disclose all relationships/activities/interests listed below that are related to the content of your manuscript. "Related" means any relation with for-profit or not-for-profit third parties whose interests may be affected by the content of the manuscript. Disclosure represents a commitment to transparency and does not necessarily indicate a bias. If you are in doubt about whether to list a relationship/activity/interest, it is preferable that you do so.**

**The following questions apply to the author's relationships/activities/interests as they relate to the current manuscript only.**

**The author's relationships/activities/interests should be defined broadly. For example, if your manuscript pertains to the epidemiology of hypertension, you should declare all relationships with manufacturers of antihypertensive medication, even if that medication is not mentioned in the manuscript.**

**In item #1 below, report all support for the work reported in this manuscript without time limit. For all other items, the time frame for disclosure is the past 36 months.**

| | | Name all entities with whom you have this relationship or indicate none (add rows as needed) | Specifications/Comments (e.g., if payments were made to you or to your institution) |
|---|---|---|---|
| **Time frame: Since the initial planning of the work** | | | |
| 1 | All support for the present manuscript (e.g., funding, provision of study materials, medical writing, article processing charges, etc.) **No time limit for this item.** | x None | |
| | | | |
| | | | |
| | | | |
| | | | |

| | | | |
|---|---|---|---|
| | | | |
| | | | |
| | | | |
| **Time frame: past 36 months** | | | |
| 2 | Grants or contracts from any entity (if not indicated in item #1 above). | x None | |
| | | | |
| | | | |
| 3 | Royalties or licenses | x None | |
| | | | |
| | | | |
| 4 | Consulting fees | x None | |
| | | | |
| | | | |
| 5 | Payment or honoraria for lectures, presentations, speakers bureaus, manuscript writing or educational events | x None | |
| | | | |
| | | | |
| 6 | Payment for expert testimony | x None | |
| | | | |
| | | | |
| 7 | Support for attending meetings and/or travel | x None | |
| | | | |
| | | | |
| 8 | Patents planned, issued or pending | x None | |
| | | | |
| | | | |
| 9 | Participation on a Data Safety Monitoring Board or Advisory | x None | |
| | | | |

| | | | |
|---|---|---|---|
| | Board | | |
| 10 | Leadership or fiduciary role in other board, society, committee or advocacy group, paid or unpaid | x None | |
| | | | |
| | | | |
| 11 | Stock or stock options | x None | |
| | | | |
| | | | |
| 12 | Receipt of equipment, materials, drugs, medical writing, gifts or other services | x None | |
| | | | |
| | | | |
| 13 | Other financial or non-financial interests | x None | |
| | | | |
| | | | |

**Please place an "X" next to the following statement to indicate your agreement:**

**_x_ I certify that I have answered every question and have not altered the wording of any of the questions on this form.**

**27.07.2021**

# ICMJE DISCLOSURE FORM

**Date:_3-AUG-2021___________________________________________________________________**
**Your Name:___Martin Laimer_____________________________________________________________**
**Manuscript Title:__Phase I/IIa clinical trial of ABCB5-positive dermal mesenchymal stem cells for recessive dystrophic epidermolysis bullosa_____________________________________________________________________________**
**Manuscript number (if known):________________________________________________________________**

**In the interest of transparency, we ask you to disclose all relationships/activities/interests listed below that are related to the content of your manuscript. "Related" means any relation with for-profit or not-for-profit third parties whose interests may be affected by the content of the manuscript. Disclosure represents a commitment to transparency and does not necessarily indicate a bias. If you are in doubt about whether to list a relationship/activity/interest, it is preferable that you do so.**

**The following questions apply to the author's relationships/activities/interests as they relate to the current manuscript only.**

**The author's relationships/activities/interests should be defined broadly. For example, if your manuscript pertains to the epidemiology of hypertension, you should declare all relationships with manufacturers of antihypertensive medication, even if that medication is not mentioned in the manuscript.**

**In item #1 below, report all support for the work reported in this manuscript without time limit. For all other items, the time frame for disclosure is the past 36 months.**

| | | **Name all entities with whom you have this relationship or indicate none (add rows as needed)** | **Specifications/Comments (e.g., if payments were made to you or to your institution)** |
|---|---|---|---|
| **Time frame: Since the initial planning of the work** | | | |
| 1 | All support for the present manuscript (e.g., funding, provision of study materials, medical writing, article processing charges, etc.) **No time limit for this item.** | ____ None | Research/Clinical Trial Support: Rheacell GmbH Co KG |
| | | | |
| | | | |
| | | | |
| | | | |
| | | | |
| | | | |
| | | | |
| **Time frame: past 36 months** | | | |
| 2 | Grants or contracts from any entity (if not indicated in item #1 above). | _X__ None | |
| | | | |
| | | | |
| 3 | Royalties or licenses | X_ None | |
| | | | |

| | | | |
|---|---|---|---|
| 4 | Consulting fees | _X_ None | |
| | | | |
| | | | |
| 5 | Payment or honoraria for lectures, presentations, speakers bureaus, manuscript writing or educational events | __X_ None | |
| | | | |
| | | | |
| 6 | Payment for expert testimony | _X_ None | |
| | | | |
| | | | |
| 7 | Support for attending meetings and/or travel | _X_ None | |
| | | | |
| | | | |
| 8 | Patents planned, issued or pending | _X__ None | |
| | | | |
| | | | |
| 9 | Participation on a Data Safety Monitoring Board or Advisory Board | _X_ None | |
| | | | |
| | | | |
| 10 | Leadership or fiduciary role in other board, society, committee or advocacy group, paid or unpaid | ____ None | Secretary General, Austrian Society of Dermatology |
| | | | |
| | | | |
| 11 | Stock or stock options | _X None | |
| | | | |
| | | | |
| 12 | Receipt of equipment, materials, drugs, medical writing, gifts or other services | _X__ None | |
| | | | |
| | | | |
| 13 | Other financial or non-financial interests | _X_ None | |
| | | | |
| | | | |

**Please place an "X" next to the following statement to indicate your agreement:**

**_X_ I certify that I have answered every question and have not altered the wording of any of the questions on this form.**

## ICMJE DISCLOSURE FORM

Date:__________________03/8/2021_______________________________________________

Your Name:___Johann W. Bauer________________________________________________

Manuscript Title:_ Phase I/IIa clinical trial of ABCB5-positive dermal mesenchymal stem cells for recessive dystrophic epidermolysis bullosa

_______________________________________________________________

Manuscript number (if known):_______________________________________________

In the interest of transparency, we ask you to disclose all relationships/activities/interests listed below that are related to the content of your manuscript. "Related" means any relation with for-profit or not-for-profit third parties whose interests may be affected by the content of the manuscript. Disclosure represents a commitment to transparency and does not necessarily indicate a bias. If you are in doubt about whether to list a relationship/activity/interest, it is preferable that you do so.

The following questions apply to the author's relationships/activities/interests as they relate to the current manuscript only.

The author's relationships/activities/interests should be defined broadly. For example, if your manuscript pertains to the epidemiology of hypertension, you should declare all relationships with manufacturers of antihypertensive medication, even if that medication is not mentioned in the manuscript.

In item #1 below, report all support for the work reported in this manuscript without time limit. For all other items, the time frame for disclosure is the past 36 months.

| | | Name all entities with whom you have this relationship or indicate none (add rows as needed) | Specifications/Comments (e.g., if payments were made to you or to your institution) |
|---|---|---|---|
| | | Time frame: Since the initial planning of the work | |
| 1 | All support for the present manuscript (e.g., funding, provision of study materials, medical writing, article processing charges, etc.) **No time limit for this item.** | ____ None | |
| | | | |
| | | | |
| | | | |
| | | | |
| | | | |
| | | | |
| | | | |
| | | Time frame: past 36 months | |
| 2 | Grants or contracts from any entity (if not indicated in item #1 above). | ____ None | |
| | | | |

| | | | |
|---|---|---|---|
| 3 | Royalties or licenses | ____ None | |
| | | | |
| | | | |
| 4 | Consulting fees | ____ None | |
| | | | |
| | | | |
| 5 | Payment or honoraria for lectures, presentations, speakers bureaus, manuscript writing or educational events | ____ None | |
| | | | |
| | | | |
| 6 | Payment for expert testimony | ____ None | Abbvie, Allmirall |
| | | | |
| | | | |
| 7 | Support for attending meetings and/or travel | ____ None | |
| | | | |
| | | | |
| 8 | Patents planned, issued or pending | ____ None | |
| | | | |
| | | | |
| 9 | Participation on a Data Safety Monitoring Board or Advisory Board | ____ None | |
| | | | |
| | | | |
| 10 | Leadership or fiduciary role in other board, society, committee or advocacy group, paid or unpaid | ____ | President Austrian Society of Dermatology and Venerology; Board Member EADV |
| | | | |
| | | | |
| 11 | Stock or stock options | ____ None | |
| | | | |
| | | | |
| 12 | Receipt of equipment, materials, drugs, medical writing, gifts or other services | ____ None | |
| | | | |
| | | | |
| 13 | Other financial or non-financial interests | ____ None | |
| | | | |
| | | | |

**Please place an "X" next to the following statement to indicate your agreement:**

__x_ **I certify that I have answered every question and have not altered the wording of any of the questions on this form.**

4.8.21

UNIKLINIKUM SALZBURG
LANDESKRANKENHAUS
Universitätsklinik für Dermatologie
und Allergologie der PMU
Vorstand:
Prim. Univ.-Prof. Dr. Johann Bauer, MBA
A-5020 Salzburg | Müllner Hauptstraße 48

## ICMJE DISCLOSURE FORM

**Date: August 2, 2021**
**Your Name: Hovnanian Alain**
**Manuscript Title:_ Phase I/IIa clinical trial of ABCB5-positive dermal mesenchymal stem cells for recessive dystrophic epidermolysis bullosa**
**Manuscript number (if known):______________________________________________________________**

**In the interest of transparency, we ask you to disclose all relationships/activities/interests listed below that are related to the content of your manuscript. "Related" means any relation with for-profit or not-for-profit third parties whose interests may be affected by the content of the manuscript. Disclosure represents a commitment to transparency and does not necessarily indicate a bias. If you are in doubt about whether to list a relationship/activity/interest, it is preferable that you do so.**

**The following questions apply to the author's relationships/activities/interests as they relate to the current manuscript only.**

**The author's relationships/activities/interests should be defined broadly. For example, if your manuscript pertains to the epidemiology of hypertension, you should declare all relationships with manufacturers of antihypertensive medication, even if that medication is not mentioned in the manuscript.**

**In item #1 below, report all support for the work reported in this manuscript without time limit. For all other items, the time frame for disclosure is the past 36 months.**

| | | **Name all entities with whom you have this relationship or indicate none (add rows as needed)** | **Specifications/Comments (e.g., if payments were made to you or to your institution)** |
|---|---|---|---|
| | | **Time frame: Since the initial planning of the work** | |
| 1 | All support for the present manuscript (e.g., funding, provision of study materials, medical writing, article processing charges, etc.) **No time limit for this item.** | __X__ None | |
| | | | |
| | | | |
| | | | |
| | | | |
| | | | |
| | | | |
| | | | |
| | | **Time frame: past 36 months** | |
| 2 | Grants or contracts from any entity (if not indicated in item #1 above). | __X__ None | |
| | | | |
| | | | |
| 3 | Royalties or licenses | __X__ None | |
| | | | |

| | | | |
|---|---|---|---|
| 4 | Consulting fees | _X___ None | |
| | | | |
| | | | |
| 5 | Payment or honoraria for lectures, presentations, speakers bureaus, manuscript writing or educational events | __X__ None | |
| | | | |
| | | | |
| 6 | Payment for expert testimony | _X___ None | |
| | | | |
| | | | |
| 7 | Support for attending meetings and/or travel | __X__ None | |
| | | | |
| | | | |
| 8 | Patents planned, issued or pending | __X__ None | |
| | | | |
| | | | |
| 9 | Participation on a Data Safety Monitoring Board or Advisory Board | __X__ None | |
| | | | |
| | | | |
| 10 | Leadership or fiduciary role in other board, society, committee or advocacy group, paid or unpaid | __X__ None | |
| | | | |
| | | | |
| 11 | Stock or stock options | __X__ None | |
| | | | |
| | | | |
| 12 | Receipt of equipment, materials, drugs, medical writing, gifts or other services | __X__ None | |
| | | | |
| | | | |
| 13 | Other financial or non-financial interests | __X__ None | |
| | | | |
| | | | |

**Please place an "X" next to the following statement to indicate your agreement:**

**X I certify that I have answered every question and have not altered the wording of any of the questions on this form.**

## ICMJE DISCLOSURE FORM

**Date:23/07/21**
**Your Name: Giovanna Zambruno**
**Manuscript Title: Phase I/IIa clinical trial of ABCB5-positive dermal mesenchymal stem cells for recessive dystrophic epidermolysis bullosa**
**Manuscript number (if known):**

**In the interest of transparency, we ask you to disclose all relationships/activities/interests listed below that are related to the content of your manuscript. "Related" means any relation with for-profit or not-for-profit third parties whose interests may be affected by the content of the manuscript. Disclosure represents a commitment to transparency and does not necessarily indicate a bias. If you are in doubt about whether to list a relationship/activity/interest, it is preferable that you do so.**

**The following questions apply to the author's relationships/activities/interests as they relate to the current manuscript only.**

**The author's relationships/activities/interests should be defined broadly. For example, if your manuscript pertains to the epidemiology of hypertension, you should declare all relationships with manufacturers of antihypertensive medication, even if that medication is not mentioned in the manuscript.**

**In item #1 below, report all support for the work reported in this manuscript without time limit. For all other items, the time frame for disclosure is the past 36 months.**

| | | Name all entities with whom you have this relationship or indicate none (add rows as needed) | Specifications/Comments (e.g., if payments were made to you or to your institution) |
|---|---|---|---|
| | | **Time frame: Since the initial planning of the work** | |
| 1 | All support for the present manuscript (e.g., funding, provision of study materials, medical writing, article processing charges, etc.) **No time limit for this item.** | __X__ None | |
| | | | |
| | | | |
| | | | |
| | | | |
| | | | |
| | | | |
| | | | |
| | | **Time frame: past 36 months** | |
| 2 | Grants or contracts from any entity (if not indicated in item #1 above). | DEBRA INTERNATIONAL | Research grant to my hospital |
| | | | |
| | | | |
| 3 | Royalties or licenses | __X__ None | |
| | | | |
| | | | |

| | | | |
|---|---|---|---|
| 4 | Consulting fees | ___X_ None | |
| 5 | Payment or honoraria for lectures, presentations, speakers bureaus, manuscript writing or educational events | __X__ None | |
| 6 | Payment for expert testimony | __x__ None | |
| 7 | Support for attending meetings and/or travel | _ RHEACELL | Travel expenses for meting attendance reimbursed to myself |
| 8 | Patents planned, issued or pending | __X__ None | |
| 9 | Participation on a Data Safety Monitoring Board or Advisory Board | DEBRA INTERNATIONAL MSAP | No fees, only reimbursement of travel/accomodation expenses to myself |
| 10 | Leadership or fiduciary role in other board, society, committee or advocacy group, paid or unpaid | ___X_ None | |
| 11 | Stock or stock options | __X__ None | |
| 12 | Receipt of equipment, materials, drugs, medical writing, gifts or other services | __X__ None | |
| 13 | Other financial or non-financial interests | ___X_ None | |

**Please place an "X" next to the following statement to indicate your agreement:**

**_X__ I certify that I have answered every question and have not altered the wording of any of the questions on this form.**

## ICMJE DISCLOSURE FORM

Date:23/07/21

Your Name: May El Hachem

Manuscript Title: Phase I/IIa clinical trial of ABCB5-positive dermal mesenchymal stem cells for recessive dystrophic epidermolysis bullosa

Manuscript number (if known):

**In the interest of transparency, we ask you to disclose all relationships/activities/interests listed below that are related to the content of your manuscript. "Related" means any relation with for-profit or not-for-profit third parties whose interests may be affected by the content of the manuscript. Disclosure represents a commitment to transparency and does not necessarily indicate a bias. If you are in doubt about whether to list a relationship/activity/interest, it is preferable that you do so.**

**The following questions apply to the author's relationships/activities/interests as they relate to the current manuscript only.**

**The author's relationships/activities/interests should be defined broadly. For example, if your manuscript pertains to the epidemiology of hypertension, you should declare all relationships with manufacturers of antihypertensive medication, even if that medication is not mentioned in the manuscript.**

**In item #1 below, report all support for the work reported in this manuscript without time limit. For all other items, the time frame for disclosure is the past 36 months.**

| | | Name all entities with whom you have this relationship or indicate none (add rows as needed) | Specifications/Comments (e.g., if payments were made to you or to your institution) |
|---|---|---|---|
| | | **Time frame: Since the initial planning of the work** | |
| 1 | All support for the present manuscript (e.g., funding, provision of study materials, medical writing, article processing charges, etc.) **No time limit for this item.** | RHEA CELL | Site non activated |
| | | | |
| | | **Time frame: past 36 months** | |
| 2 | Grants or contracts from any entity (if not indicated in item #1 above). | AMRYT | Contract with my institution for a clinical trial |
| | | | |
| 3 | Royalties or licenses | __X__ None | |
| | | | |

| | | | |
|---|---|---|---|
| 4 | Consulting fees | ___X_ None | |
| | | | |
| | | | |
| 5 | Payment or honoraria for lectures, presentations, speakers bureaus, manuscript writing or educational events | __X__ None | |
| | | | |
| | | | |
| 6 | Payment for expert testimony | __x__ None | |
| | | | |
| | | | |
| 7 | Support for attending meetings and/or travel | _X___ None | |
| | | | |
| | | | |
| 8 | Patents planned, issued or pending | __X__ None | |
| | | | |
| | | | |
| 9 | Participation on a Data Safety Monitoring Board or Advisory Board | __X__ None | |
| | | | |
| | | | |
| 10 | Leadership or fiduciary role in other board, society, committee or advocacy group, paid or unpaid | ___X_ None | |
| | | | |
| | | | |
| 11 | Stock or stock options | __X__ None | |
| | | | |
| | | | |
| 12 | Receipt of equipment, materials, drugs, medical writing, gifts or other services | __X__ None | |
| | | | |
| | | | |
| 13 | Other financial or non-financial interests | ___X_ None | |
| | | | |
| | | | |

**Please place an "X" next to the following statement to indicate your agreement:**

**_X__ I certify that I have answered every question and have not altered the wording of any of the questions on this form.**

Dott.ssa EL HACHEM MAY
RESPONSABILE DELLA STRUTTURA
COMPLESSA DI DERMATOLOGIA

ICMJE DISCLOSURE FORM

Date: Wednesday 4th August 2021

Your Name: Bourrat Emmanuelle
Manuscript Title:_ Phase I/IIa clinical trial of ABCB5-positive dermal mesenchymal stem cells for recessive dystrophic epidermolysis bullosa
Manuscript number (if known) : unknown

**In the interest of transparency, we ask you to disclose all relationships/activities/interests listed below that are related to the content of your manuscript. "Related" means any relation with for-profit or not-for-profit third parties whose interests may be affected by the content of the manuscript. Disclosure represents a commitment to transparency and does not necessarily indicate a bias. If you are in doubt about whether to list a relationship/activity/interest, it is preferable that you do so.**

**The following questions apply to the author's relationships/activities/interests as they relate to the <u>current manuscript only</u>.**

**The author's relationships/activities/interests should be <u>defined broadly</u>. For example, if your manuscript pertains to the epidemiology of hypertension, you should declare all relationships with manufacturers of antihypertensive medication, even if that medication is not mentioned in the manuscript.**

**In item #1 below, report all support for the work reported in this manuscript without time limit. For all other items, the time frame for disclosure is the past 36 months.**

| | | **Name all entities with whom you have this relationship or indicate none (add rows as needed)** | **Specifications/Comments (e.g., if payments were made to you or to your institution)** |
|---|---|---|---|
| | | **Time frame: Since the initial planning of the work** | |
| 1 | All support for the present manuscript (e.g., funding, provision of study materials, medical writing, article processing charges, etc.) **No time limit for this item.** | ____ None | |
| | | | |
| | | | |
| | | | |
| | | | |
| | | | |
| | | | |
| | | **Time frame: past 36 months** | |
| 2 | Grants or contracts from any entity (if not indicated in item #1 above). | ____ None | |
| | | | |
| | | | |
| 3 | Royalties or licenses | ____ None | |
| | | | |

| | | | |
|---|---|---|---|
| 4 | Consulting fees | ____ None | |
| | | | |
| | | | |
| 5 | Payment or honoraria for lectures, presentations, speakers bureaus, manuscript writing or educational events | ____ None | |
| | | | |
| | | | |
| 6 | Payment for expert testimony | ____ None | |
| | | | |
| | | | |
| 7 | Support for attending meetings and/or travel | ____ None | |
| | | | |
| | | | |
| 8 | Patents planned, issued or pending | ____ None | |
| | | | |
| | | | |
| 9 | Participation on a Data Safety Monitoring Board or Advisory Board | ____ None | |
| | | | |
| | | | |
| 10 | Leadership or fiduciary role in other board, society, committee or advocacy group, paid or unpaid | ____ None | |
| | | | |
| | | | |
| 11 | Stock or stock options | ____ None | |
| | | | |
| | | | |
| 12 | Receipt of equipment, materials, drugs, medical writing, gifts or other services | ____ None | |
| | | | |
| | | | |
| 13 | Other financial or non-financial interests | ____ None | |
| | | | |
| | | | |

**Please place an "X" next to the following statement to indicate your agreement:**

**_X_ I certify that I have answered every question and have not altered the wording of any of the questions on this form.**

# ICMJE DISCLOSURE FORM

**Date:** Wednesday 4th August 2021
**Your Name:** Maria Papanikolaou
**Manuscript Title:** Phase I/IIa clinical trial of ABCB5-positive dermal mesenchymal stem cells for recessive dystrophic epidermolysis bullosa
**Manuscript number (if known):** not known

**In the interest of transparency, we ask you to disclose all relationships/activities/interests listed below that are related to the content of your manuscript. "Related" means any relation with for-profit or not-for-profit third parties whose interests may be affected by the content of the manuscript. Disclosure represents a commitment to transparency and does not necessarily indicate a bias. If you are in doubt about whether to list a relationship/activity/interest, it is preferable that you do so.**

**The following questions apply to the author's relationships/activities/interests as they relate to the current manuscript only.**

**The author's relationships/activities/interests should be defined broadly. For example, if your manuscript pertains to the epidemiology of hypertension, you should declare all relationships with manufacturers of antihypertensive medication, even if that medication is not mentioned in the manuscript.**

**In item #1 below, report all support for the work reported in this manuscript without time limit. For all other items, the time frame for disclosure is the past 36 months.**

| | | Name all entities with whom you have this relationship or indicate none (add rows as needed) | Specifications/Comments (e.g., if payments were made to you or to your institution) |
|---|---|---|---|
| | | **Time frame: Since the initial planning of the work** | |
| 1 | All support for the present manuscript (e.g., funding, provision of study materials, medical writing, article processing charges, etc.) No time limit for this item. | ____ None | |
| | | | |
| | | | |
| | | | |
| | | | |
| | | | |
| | | | |
| | | | |
| | | **Time frame: past 36 months** | |
| 2 | Grants or contracts from any entity (if not indicated in item #1 above). | ____ None | |
| | | | |
| | | | |
| 3 | Royalties or licenses | ____ None | |
| | | | |

| | | | |
|---|---|---|---|
| | | | |
| 4 | Consulting fees | ____ None | |
| | | | |
| | | | |
| 5 | Payment or honoraria for lectures, presentations, speakers bureaus, manuscript writing or educational events | ____ None | |
| | | | |
| | | | |
| 6 | Payment for expert testimony | ____ None | |
| | | | |
| | | | |
| 7 | Support for attending meetings and/or travel | ____ None | |
| | | | |
| | | | |
| 8 | Patents planned, issued or pending | ____ None | |
| | | | |
| | | | |
| 9 | Participation on a Data Safety Monitoring Board or Advisory Board | ____ None | |
| | | | |
| | | | |
| 10 | Leadership or fiduciary role in other board, society, committee or advocacy group, paid or unpaid | ____ None | |
| | | | |
| | | | |
| 11 | Stock or stock options | ____ None | |
| | | | |
| | | | |
| 12 | Receipt of equipment, materials, drugs, medical writing, gifts or other services | ____ None | |
| | | | |
| | | | |
| 13 | Other financial or non-financial interests | ____ None | |
| | | | |
| | | | |

Please place an "X" next to the following statement to indicate your agreement:

X I certify that I have answered every question and have not altered the wording of any of the questions on this form.

**ICMJE DISCLOSURE FORM**

**Date:** 3/8/21
**Your Name:** Gabriela Petrof
**Manuscript Title:** Phase I/IIa clinical trial of ABCB5+ dermal MSCs for RDEB
**Manuscript number (if known):**__________

**In the interest of transparency, we ask you to disclose all relationships/activities/interests listed below that are related to the content of your manuscript. "Related" means any relation with for-profit or not-for-profit third parties whose interests may be affected by the content of the manuscript. Disclosure represents a commitment to transparency and does not necessarily indicate a bias. If you are in doubt about whether to list a relationship/activity/interest, it is preferable that you do so.**

**The following questions apply to the author's relationships/activities/interests as they relate to the current manuscript only.**

**The author's relationships/activities/interests should be defined broadly. For example, if your manuscript pertains to the epidemiology of hypertension, you should declare all relationships with manufacturers of antihypertensive medication, even if that medication is not mentioned in the manuscript.**

**In item #1 below, report all support for the work reported in this manuscript without time limit. For all other items, the time frame for disclosure is the past 36 months.**

| | | Name all entities with whom you have this relationship or indicate none (add rows as needed) | Specifications/Comments (e.g., if payments were made to you or to your institution) |
|---|---|---|---|
| | | Time frame: Since the initial planning of the work | |
| 1 | All support for the present manuscript (e.g., funding, provision of study materials, medical writing, article processing charges, etc.) **No time limit for this item.** | ☒ None<br>Acted as sub-investigator collected and reviewed data No directly paid work | |
| | | Time frame: past 36 months | |
| 2 | Grants or contracts from any entity (if not indicated in item #1 above). | ✓ None | |
| 3 | Royalties or licenses | ✓ None | |

| | | | |
|---|---|---|---|
| 4 | Consulting fees | ✓ None | |
| 5 | Payment or honoraria for lectures, presentations, speakers bureaus, manuscript writing or educational events | ✓ None | |
| 6 | Payment for expert testimony | ✓ None | |
| 7 | Support for attending meetings and/or travel | ✓ None | |
| 8 | Patents planned, issued or pending | ✓ None | |
| 9 | Participation on a Data Safety Monitoring Board or Advisory Board | ✓ None | |
| 10 | Leadership or fiduciary role in other board, society, committee or advocacy group, paid or unpaid | ✓ None | |
| 11 | Stock or stock options | ✓ None | |
| 12 | Receipt of equipment, materials, drugs, medical writing, gifts or other services | ✓ None | |
| 13 | Other financial or non-financial interests | ✓ None | |

**Please place an "X" next to the following statement to indicate your agreement:**

X **I certify that I have answered every question and have not altered the wording of any of the questions on this form.**

## ICMJE DISCLOSURE FORM

**Date:** 23-JUL-2021
**Your Name:** Dr. Sophie Kitzmüller
**Manuscript Title:** Phase I/IIa clinical trial of ABCB5-positive dermal mesenchymal stem cells for recessive dystrophic epidermolysis bullosa
**Manuscript number (if known):**________________________________________

**In the interest of transparency, we ask you to disclose all relationships/activities/interests listed below that are related to the content of your manuscript. "Related" means any relation with for-profit or not-for-profit third parties whose interests may be affected by the content of the manuscript. Disclosure represents a commitment to transparency and does not necessarily indicate a bias. If you are in doubt about whether to list a relationship/activity/interest, it is preferable that you do so.**

**The following questions apply to the author's relationships/activities/interests as they relate to the current manuscript only.**

**The author's relationships/activities/interests should be defined broadly. For example, if your manuscript pertains to the epidemiology of hypertension, you should declare all relationships with manufacturers of antihypertensive medication, even if that medication is not mentioned in the manuscript.**

**In item #1 below, report all support for the work reported in this manuscript without time limit. For all other items, the time frame for disclosure is the past 36 months.**

| | | Name all entities with whom you have this relationship or indicate none (add rows as needed) | Specifications/Comments (e.g., if payments were made to you or to your institution) |
|---|---|---|---|
| | | **Time frame: Since the initial planning of the work** | |
| 1 | All support for the present manuscript (e.g., funding, provision of study materials, medical writing, article processing charges, etc.) **No time limit for this item.** | ____ None | |
| | | | |
| | | **Time frame: past 36 months** | |
| 2 | Grants or contracts from any entity (if not indicated in item #1 above). | DEBRA Austria | Salary paid by DEBRA Austria |
| | | | |
| 3 | Royalties or licenses | ____ None | |
| | | | |

| | | | |
|---|---|---|---|
| | | | |
| 4 | Consulting fees | ____ None | |
| 5 | Payment or honoraria for lectures, presentations, speakers bureaus, manuscript writing or educational events | Amryt Pharma Plc<br>Mölnlycke Health Care AB | Honoraria for educational talks |
| 6 | Payment for expert testimony | ____ None | |
| 7 | Support for attending meetings and/or travel | ____ None | |
| 8 | Patents planned, issued or pending | ____ None | |
| 9 | Participation on a Data Safety Monitoring Board or Advisory Board | ____ None | |
| 10 | Leadership or fiduciary role in other board, society, committee or advocacy group, paid or unpaid | ____ None | |
| 11 | Stock or stock options | ____ None | |
| 12 | Receipt of equipment, materials, drugs, medical writing, gifts or other services | ____ None | |
| 13 | Other financial or non-financial interests | ____ None | |

**Please place an "X" next to the following statement to indicate your agreement:**

X **I certify that I have answered every question and have not altered the wording of any of the questions on this form.**

**ICMJE DISCLOSURE FORM**

**Date:**_______22 July 2021____________________________________________________________________
**Your Name**:_____Christen L. Ebens, MD MPH_________________________________________________________
**Manuscript Title**: Phase I/IIa clinical trial of ABCB5-positive dermal mesenchymal stem cells for recessive dystrophic epidermolysis bullosa______________________
**Manuscript number (if known):**____ 151922-JCI-CMED_________________________________________________

**In the interest of transparency, we ask you to disclose all relationships/activities/interests listed below that are related to the content of your manuscript. "Related" means any relation with for-profit or not-for-profit third parties whose interests may be affected by the content of the manuscript. Disclosure represents a commitment to transparency and does not necessarily indicate a bias. If you are in doubt about whether to list a relationship/activity/interest, it is preferable that you do so.**

**The following questions apply to the author's relationships/activities/interests as they relate to the current manuscript only.**

**The author's relationships/activities/interests should be defined broadly. For example, if your manuscript pertains to the epidemiology of hypertension, you should declare all relationships with manufacturers of antihypertensive medication, even if that medication is not mentioned in the manuscript.**

**In item #1 below, report all support for the work reported in this manuscript without time limit. For all other items, the time frame for disclosure is the past 36 months.**

| | | **Name all entities with whom you have this relationship or indicate none (add rows as needed)** | **Specifications/Comments (e.g., if payments were made to you or to your institution)** |
|---|---|---|---|
| | | **Time frame: Since the initial planning of the work** | |
| 1 | All support for the present manuscript (e.g., funding, provision of study materials, medical writing, article processing charges, etc.) **No time limit for this item.** | __X__ None | |
| | | | |
| | | | |
| | | | |
| | | | |
| | | | |
| | | | |
| | | | |
| | | **Time frame: past 36 months** | |
| 2 | Grants or contracts from any entity (if not indicated in item #1 above). | ____ None | Contract with sponsor (Rheacell Gmbh & Co) for conduct of clinical trial of allo-APZ2-EB MSCs in RDEB, outcomes described in this manuscript |
| | | | |
| | | | |
| 3 | Royalties or licenses | _X___ None | |

| | | | |
|---|---|---|---|
| | | | |
| | | | |
| 4 | Consulting fees | __X__ None | |
| | | | |
| | | | |
| 5 | Payment or honoraria for lectures, presentations, speakers bureaus, manuscript writing or educational events | _X___ None | |
| | | | |
| | | | |
| 6 | Payment for expert testimony | __X__ None | |
| | | | |
| | | | |
| 7 | Support for attending meetings and/or travel | __X__ None | |
| | | | |
| | | | |
| 8 | Patents planned, issued or pending | _X___ None | |
| | | | |
| | | | |
| 9 | Participation on a Data Safety Monitoring Board or Advisory Board | __X__ None | |
| | | | |
| | | | |
| 10 | Leadership or fiduciary role in other board, society, committee or advocacy group, paid or unpaid | _X___ None | |
| | | | |
| | | | |
| 11 | Stock or stock options | _X___ None | |
| | | | |
| | | | |
| 12 | Receipt of equipment, materials, drugs, medical writing, gifts or other services | _X___ None | |
| | | | |
| | | | |
| 13 | Other financial or non-financial interests | _X___ None | |
| | | | |
| | | | |

**Please place an "X" next to the following statement to indicate your agreement:**

**__X_ I certify that I have answered every question and have not altered the wording of any of the questions on this form.**

## ICMJE DISCLOSURE FORM

**Date: 07/23/2021**
**Your Name: Markus H. Frank**
**Manuscript Title: Phase I/IIa clinical trial of ABCB5-positive dermal mesenchymal stem cells for recessive dystrophic epidermolysis bullosa**
**Manuscript number (if known): 151922-INS-CMED-TR-2**

**In the interest of transparency, we ask you to disclose all relationships/activities/interests listed below that are related to the content of your manuscript. "Related" means any relation with for-profit or not-for-profit third parties whose interests may be affected by the content of the manuscript. Disclosure represents a commitment to transparency and does not necessarily indicate a bias. If you are in doubt about whether to list a relationship/activity/interest, it is preferable that you do so.**

**The following questions apply to the author's relationships/activities/interests as they relate to the current manuscript only.**

**The author's relationships/activities/interests should be defined broadly. For example, if your manuscript pertains to the epidemiology of hypertension, you should declare all relationships with manufacturers of antihypertensive medication, even if that medication is not mentioned in the manuscript.**

**In item #1 below, report all support for the work reported in this manuscript without time limit. For all other items, the time frame for disclosure is the past 36 months.**

| | | **Name all entities with whom you have this relationship or indicate none (add rows as needed)** | **Specifications/Comments (e.g., if payments were made to you or to your institution)** |
|---|---|---|---|
| **Time frame: Since the initial planning of the work** | | | |
| 1 | All support for the present manuscript (e.g., funding, provision of study materials, medical writing, article processing charges, etc.) **No time limit for this item.** | ____ None | |
| | | Ticeba GmbH | Paid Scientific Advisor |
| | | Rheacell GmbH & Co KG | Scientific Advisor, Past Corporate Sponsored Research Support |
| | | | |
| | | | |
| | | | |
| | | | |
| | | | |
| **Time frame: past 36 months** | | | |
| 2 | Grants or contracts from any entity (if not indicated in item #1 above). | ____ None<br>NIH/NEI | RO1EY025794 and R24EY028767 |
| | | | |
| | | | |
| 3 | Royalties or licenses | ____ None<br>Patent Royalties | MHF is inventor or co-inventor of US and international patents assigned to Brigham and Women's Hospital and/or Boston Children's Hospital, Boston, MA, USA, |

| | | | |
|---|---|---|---|
| | | | licensed to TICEBA GmbH, Heidelberg, Germany, and RHEACELL GmbH & Co. KG, Heidelberg, Germany. |
| | | | |
| | | | |
| 4 | Consulting fees | ____ None<br>Ticeba GmbH | Scientific Advisor |
| | | | |
| | | | |
| 5 | Payment or honoraria for lectures, presentations, speakers bureaus, manuscript writing or educational events | __x__ None | |
| | | | |
| | | | |
| 6 | Payment for expert testimony | __x__ None | |
| | | | |
| | | | |
| 7 | Support for attending meetings and/or travel | __x__ None | |
| | | | |
| | | | |
| 8 | Patents planned, issued or pending | ____ None<br>Patents | MHF is inventor or co-inventor of US and international patents assigned to Brigham and Women's Hospital and/or Boston Children's Hospital, Boston, MA, USA, licensed to TICEBA GmbH, Heidelberg, Germany, and RHEACELL GmbH & Co. KG, Heidelberg, Germany. |
| | | | |
| | | | |
| 9 | Participation on a Data Safety Monitoring Board or Advisory Board | ____ None<br>Ticeba GmbH | Scientific Advisory Board |
| | | Rheacell GmbH & Co KG | Scientific Advisory Board |
| | | | |
| 10 | Leadership or fiduciary role in other board, society, committee or advocacy group, paid or unpaid | _x___ None | |
| | | | |
| | | | |
| 11 | Stock or stock options | __x__ None | |
| | | | |
| | | | |
| 12 | Receipt of equipment, materials, drugs, medical writing, gifts or other services | __x__ None | |
| | | | |
| | | | |
| 13 | Other financial or non-financial interests | _x___ None | |
| | | | |
| | | | |

**Please place an "X" next to the following statement to indicate your agreement:**

**_x__ I certify that I have answered every question and have not altered the wording of any of the questions on this form.**

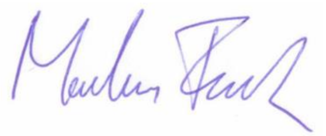

Markus Frank, M.D.

# ICMJE DISCLOSURE FORM

**Date:__________07/26/2021_____________________________________________________**
**Your Name:___Natasha Frank ________________________________________________________**
**Manuscript Title:____ Phase I/IIa clinical trial of ABCB5-positive dermal mesenchymal stem cells for recessive dystrophic epidermolysis bullosa**
**Manuscript number (if known): 151922-INS-CMED-TR-2**

**In the interest of transparency, we ask you to disclose all relationships/activities/interests listed below that are related to the content of your manuscript. "Related" means any relation with for-profit or not-for-profit third parties whose interests may be affected by the content of the manuscript. Disclosure represents a commitment to transparency and does not necessarily indicate a bias. If you are in doubt about whether to list a relationship/activity/interest, it is preferable that you do so.**

**The following questions apply to the author's relationships/activities/interests as they relate to the current manuscript only.**

**The author's relationships/activities/interests should be defined broadly. For example, if your manuscript pertains to the epidemiology of hypertension, you should declare all relationships with manufacturers of antihypertensive medication, even if that medication is not mentioned in the manuscript.**

**In item #1 below, report all support for the work reported in this manuscript without time limit. For all other items, the time frame for disclosure is the past 36 months.**

| | | **Name all entities with whom you have this relationship or indicate none (add rows as needed)** | **Specifications/Comments (e.g., if payments were made to you or to your institution)** |
|---|---|---|---|
| | | **Time frame: Since the initial planning of the work** | |
| 1 | All support for the present manuscript (e.g., funding, provision of study materials, medical writing, article processing charges, etc.) **No time limit for this item.** | __x__ None | |
| | | | |
| | | | |
| | | | |
| | | | |
| | | | |
| | | | |
| | | | |
| | | **Time frame: past 36 months** | |
| 2 | Grants or contracts from any entity (if not indicated in item #1 above). | ____ None<br>NIH/NEI | RO1EY025794 and R24EY028767 |
| | | | |
| | | | |
| 3 | Royalties or licenses | ____ None<br>Patent Royalties | NYF is inventor or co-inventor of US and international patents assigned to Brigham and Women's Hospital and/or Boston Children's Hospital, Boston, MA, USA, |

| | | | |
|---|---|---|---|
| | | | licensed to TICEBA GmbH, Heidelberg, Germany, and RHEACELL GmbH & Co. KG, Heidelberg, Germany. |
| | | | |
| | | | |
| 4 | Consulting fees | __x__ None | |
| | | | |
| | | | |
| 5 | Payment or honoraria for lectures, presentations, speakers bureaus, manuscript writing or educational events | __x__ None | |
| | | | |
| | | | |
| 6 | Payment for expert testimony | __x__ None | |
| | | | |
| | | | |
| 7 | Support for attending meetings and/or travel | __x__ None | |
| | | | |
| | | | |
| 8 | Patents planned, issued or pending | ____ None<br>Patents | NYF is inventor or co-inventor of US and international patents assigned to Brigham and Women's Hospital and/or Boston Children's Hospital, Boston, MA, USA, licensed to TICEBA GmbH, Heidelberg, Germany, and RHEACELL GmbH & Co. KG, Heidelberg, Germany. |
| | | | |
| | | | |
| 9 | Participation on a Data Safety Monitoring Board or Advisory Board | __x__ None | |
| | | | |
| | | | |
| 10 | Leadership or fiduciary role in other board, society, committee or advocacy group, paid or unpaid | __x__ None | |
| | | | |
| | | | |
| 11 | Stock or stock options | __x__ None | |
| | | | |
| | | | |
| 12 | Receipt of equipment, materials, drugs, medical writing, gifts or other services | __x__ None | |
| | | | |
| | | | |
| 13 | Other financial or non-financial interests | _x___ None | |
| | | | |
| | | | |

**Please place an "X" next to the following statement to indicate your agreement:**

**_x__ I certify that I have answered every question and have not altered the wording of any of the questions on this form.**

Natasha Frank

Natasha Frank, M.D.

## ICMJE DISCLOSURE FORM

**Date:** 27.07.2021
**Your Name:** Christoph Ganss
**Manuscript Title:** Phase I/IIa clinical trial of ABCB5-positive dermal mesenchymal stem cells for recessive dystrophic epidermolysis bullosa
**Manuscript number (if known):** 151922-INS-CMED-RV-3

**In the interest of transparency, we ask you to disclose all relationships/activities/interests listed below that are related to the content of your manuscript. "Related" means any relation with for-profit or not-for-profit third parties whose interests may be affected by the content of the manuscript. Disclosure represents a commitment to transparency and does not necessarily indicate a bias. If you are in doubt about whether to list a relationship/activity/interest, it is preferable that you do so.**

**The following questions apply to the author's relationships/activities/interests as they relate to the current manuscript only.**

**The author's relationships/activities/interests should be defined broadly. For example, if your manuscript pertains to the epidemiology of hypertension, you should declare all relationships with manufacturers of antihypertensive medication, even if that medication is not mentioned in the manuscript.**

**In item #1 below, report all support for the work reported in this manuscript without time limit. For all other items, the time frame for disclosure is the past 36 months.**

| | | **Name all entities with whom you have this relationship or indicate none (add rows as needed)** | **Specifications/Comments (e.g., if payments were made to you or to your institution)** |
|---|---|---|---|
| **Time frame: Since the initial planning of the work** | | | |
| 1 | All support for the present manuscript (e.g., funding, provision of study materials, medical writing, article processing charges, etc.) **No time limit for this item.** | X None | |
| | | | |
| | | | |
| | | | |
| | | | |
| | | | |
| | | | |
| | | | |
| **Time frame: past 36 months** | | | |
| 2 | Grants or contracts from any entity (if not indicated in item #1 above). | X None | |
| | | | |
| | | | |
| 3 | Royalties or licenses | X None | |
| | | | |

| | | | |
|---|---|---|---|
| 4 | Consulting fees | x None | |
| | | | |
| | | | |
| 5 | Payment or honoraria for lectures, presentations, speakers bureaus, manuscript writing or educational events | X None | |
| | | | |
| | | | |
| 6 | Payment for expert testimony | X None | |
| | | | |
| | | | |
| 7 | Support for attending meetings and/or travel | X None | |
| | | | |
| | | | |
| 8 | Patents planned, issued or pending | X None | |
| | | | |
| | | | |
| 9 | Participation on a Data Safety Monitoring Board or Advisory Board | X None | |
| | | | |
| | | | |
| 10 | Leadership or fiduciary role in other board, society, committee or advocacy group, paid or unpaid | X None | |
| | | | |
| | | | |
| 11 | Stock or stock options | X None | |
| | | | |
| | | | |
| 12 | Receipt of equipment, materials, drugs, medical writing, gifts or other services | X None | |
| | | | |
| | | | |
| 13 | Other financial or non-financial interests | TICEBA GmbH | CEO |
| | | RHEACELL GmbH & Co. KG | CEO |
| | | | |

**Please place an "X" next to the following statement to indicate your agreement:**

**X I certify that I have answered every question and have not altered the wording of any of the questions on this form.**

## ICMJE DISCLOSURE FORM

Date: 22.7.202[illegible]
Your Name: ANNA MARTINEZ
Manuscript Title: Phase I/II [illegible] trial data [illegible] MSC.
Manuscript number (if known): ______

In the interest of transparency, we ask you to disclose all relationships/activities/interests listed below that are related to the content of your manuscript. "Related" means any relation with for-profit or not-for-profit third parties whose interests may be affected by the content of the manuscript. Disclosure represents a commitment to transparency and does not necessarily indicate a bias. If you are in doubt about whether to list a relationship/activity/interest, it is preferable that you do so.

The following questions apply to the author's relationships/activities/interests as they relate to the current manuscript only.

The author's relationships/activities/interests should be defined broadly. For example, if your manuscript pertains to the epidemiology of hypertension, you should declare all relationships with manufacturers of antihypertensive medication, even if that medication is not mentioned in the manuscript.

In item #1 below, report all support for the work reported in this manuscript without time limit. For all other items, the time frame for disclosure is the past 36 months.

| | | Name all entities with whom you have this relationship or indicate none (add rows as needed) | Specifications/Comments (e.g., if payments were made to you or to your institution) |
|---|---|---|---|
| | | **Time frame: Since the initial planning of the work** | |
| 1 | All support for the present manuscript (e.g., funding, provision of study materials, medical writing, article processing charges, etc.) **No time limit for this item.** | X None ✓ | |
| | | **Time frame: past 36 months** | |
| 2 | Grants or contracts from any entity (if not indicated in item #1 above). | X None ✓ | |
| 3 | Royalties or licenses | X None ✓ | |

| | | | |
|---|---|---|---|
| 4 | Consulting fees | X None | |
| 5 | Payment or honoraria for lectures, presentations, speakers bureaus, manuscript writing or educational events | X None | |
| 6 | Payment for expert testimony | X None | |
| 7 | Support for attending meetings and/or travel | X None | |
| 8 | Patents planned, issued or pending | X None | |
| 9 | Participation on a Data Safety Monitoring Board or Advisory Board | X None | |
| 10 | Leadership or fiduciary role in other board, society, committee or advocacy group, paid or unpaid | X None | |
| 11 | Stock or stock options | X None | |
| 12 | Receipt of equipment, materials, drugs, medical writing, gifts or other services | X None | |
| 13 | Other financial or non-financial interests | X None | |

**Please place an "X" next to the following statement to indicate your agreement:**

**___ I certify that I have answered every question and have not altered the wording of any of the questions on this form.**

## ICMJE DISCLOSURE FORM

**Date:** 05.08.2021
**Your Name:** Prof. Dr. John McGrath
**Manuscript Title:** Phase I/IIa clinical trial of ABCB5-positive dermal mesenchymal stem cells for recessive dystrophic epidermolysis bullosa
**Manuscript number (if known):** 151922-INS-CMED-RV-3

**In the interest of transparency, we ask you to disclose all relationships/activities/interests listed below that are related to the content of your manuscript. "Related" means any relation with for-profit or not-for-profit third parties whose interests may be affected by the content of the manuscript. Disclosure represents a commitment to transparency and does not necessarily indicate a bias. If you are in doubt about whether to list a relationship/activity/interest, it is preferable that you do so.**

**The following questions apply to the author's relationships/activities/interests as they relate to the current manuscript only.**

**The author's relationships/activities/interests should be defined broadly. For example, if your manuscript pertains to the epidemiology of hypertension, you should declare all relationships with manufacturers of antihypertensive medication, even if that medication is not mentioned in the manuscript.**

**In item #1 below, report all support for the work reported in this manuscript without time limit. For all other items, the time frame for disclosure is the past 36 months.**

| | | **Name all entities with whom you have this relationship or indicate none (add rows as needed)** | **Specifications/Comments (e.g., if payments were made to you or to your institution)** |
|---|---|---|---|
| | | **Time frame: Since the initial planning of the work** | |
| 1 | All support for the present manuscript (e.g., funding, provision of study materials, medical writing, article processing charges, etc.) **No time limit for this item.** | None<br>x | |
| | | | |
| | | | |
| | | | |
| | | | |
| | | | |
| | | | |
| | | | |
| | | **Time frame: past 36 months** | |
| 2 | Grants or contracts from any entity (if not indicated in item #1 above). | None<br>x | |
| | | | |
| | | | |
| 3 | Royalties or licenses | None<br>x | |
| | | | |

| | | | |
|---|---|---|---|
| 4 | Consulting fees | None<br>x | |
| | | | |
| | | | |
| 5 | Payment or honoraria for lectures, presentations, speakers bureaus, manuscript writing or educational events | None<br>x | |
| | | | |
| | | | |
| 6 | Payment for expert testimony | None<br>x | |
| | | | |
| | | | |
| 7 | Support for attending meetings and/or travel | None<br>x | |
| | | | |
| | | | |
| 8 | Patents planned, issued or pending | None<br>x | |
| | | | |
| | | | |
| 9 | Participation on a Data Safety Monitoring Board or Advisory Board | None<br>x | |
| | | | |
| | | | |
| 10 | Leadership or fiduciary role in other board, society, committee or advocacy group, paid or unpaid | None<br>x | |
| | | | |
| | | | |
| 11 | Stock or stock options | None<br>x | |
| | | | |
| | | | |
| 12 | Receipt of equipment, materials, drugs, medical writing, gifts or other services | None<br>x | |
| | | | |
| | | | |
| 13 | Other financial or non-financial interests | None<br>x | |
| | | | |
| | | | |

**Please place an "X" next to the following statement to indicate your agreement:**

**X I certify that I have answered every question and have not altered the wording of any of the questions on this form.**

John McGrath 5 August 2021

## ICMJE DISCLOSURE FORM

Date: 22 July 2021
Your Name: JAKUB TOLAR
Manuscript Title: Phase I/IIa clinical trial of ABCB5+ cells for RDEB.
Manuscript number (if known):

**In the interest of transparency, we ask you to disclose all relationships/activities/interests listed below that are related to the content of your manuscript. "Related" means any relation with for-profit or not-for-profit third parties whose interests may be affected by the content of the manuscript. Disclosure represents a commitment to transparency and does not necessarily indicate a bias. If you are in doubt about whether to list a relationship/activity/interest, it is preferable that you do so.**

**The following questions apply to the author's relationships/activities/interests as they relate to the current manuscript only.**

**The author's relationships/activities/interests should be defined broadly. For example, if your manuscript pertains to the epidemiology of hypertension, you should declare all relationships with manufacturers of antihypertensive medication, even if that medication is not mentioned in the manuscript.**

**In item #1 below, report all support for the work reported in this manuscript without time limit. For all other items, the time frame for disclosure is the past 36 months.**

| | | **Name all entities with whom you have this relationship or indicate none (add rows as needed)** | **Specifications/Comments (e.g., if payments were made to you or to your institution)** |
|---|---|---|---|
| | | **Time frame: Since the initial planning of the work** | |
| 1 | All support for the present manuscript (e.g., funding, provision of study materials, medical writing, article processing charges, etc.) **No time limit for this item.** | ___ None | |
| | | R01-AR063070 | |
| | | | |
| | | **Time frame: past 36 months** | |
| 2 | Grants or contracts from any entity (if not indicated in item #1 above). | ✓ None | |
| | | | |
| 3 | Royalties or licenses | ✓ None | |
| | | | |

| | | | |
|---|---|---|---|
| 4 | Consulting fees | ___ None<br>For TICEBA. | |
| 5 | Payment or honoraria for lectures, presentations, speakers bureaus, manuscript writing or educational events | ✓ None | |
| 6 | Payment for expert testimony | ✓ None | |
| 7 | Support for attending meetings and/or travel | ✓ None | |
| 8 | Patents planned, issued or pending | ✓ None | |
| 9 | Participation on a Data Safety Monitoring Board or Advisory Board | ___ None<br>TICEBA DSMB member. | |
| 10 | Leadership or fiduciary role in other board, society, committee or advocacy group, paid or unpaid | ✓ None | |
| 11 | Stock or stock options | ✓ None | |
| 12 | Receipt of equipment, materials, drugs, medical writing, gifts or other services | ✓ None | |
| 13 | Other financial or non-financial interests | ✓ None | |

**Please place an "X" next to the following statement to indicate your agreement:**

✓ **I certify that I have answered every question and have not altered the wording of any of the questions on this form.**

## ICMJE DISCLOSURE FORM

**Date:** 22.07.2021
**Your Name:** Mark Andreas Kluth
**Manuscript Title:** Phase I/IIa clinical trial of ABCB5-positive dermal mesenchymal stem cells for recessive dystrophic epidermolysis bullosa
**Manuscript number (if known):** 151922-INS-CMED-RV-3

**In the interest of transparency, we ask you to disclose all relationships/activities/interests listed below that are related to the content of your manuscript. "Related" means any relation with for-profit or not-for-profit third parties whose interests may be affected by the content of the manuscript. Disclosure represents a commitment to transparency and does not necessarily indicate a bias. If you are in doubt about whether to list a relationship/activity/interest, it is preferable that you do so.**

**The following questions apply to the author's relationships/activities/interests as they relate to the current manuscript only.**

**The author's relationships/activities/interests should be defined broadly. For example, if your manuscript pertains to the epidemiology of hypertension, you should declare all relationships with manufacturers of antihypertensive medication, even if that medication is not mentioned in the manuscript.**

**In item #1 below, report all support for the work reported in this manuscript without time limit. For all other items, the time frame for disclosure is the past 36 months.**

| | | **Name all entities with whom you have this relationship or indicate none (add rows as needed)** | **Specifications/Comments (e.g., if payments were made to you or to your institution)** |
|---|---|---|---|
| **Time frame: Since the initial planning of the work** | | | |
| 1 | All support for the present manuscript (e.g., funding, provision of study materials, medical writing, article processing charges, etc.) **No time limit for this item.** | X None | |
| | | | |
| | | | |
| | | | |
| | | | |
| | | | |
| | | | |
| | | | |
| **Time frame: past 36 months** | | | |
| 2 | Grants or contracts from any entity (if not indicated in item #1 above). | X None | |
| | | | |
| | | | |
| 3 | Royalties or licenses | X None | |
| | | | |

| | | | |
|---|---|---|---|
| 4 | Consulting fees | x None | |
| | | | |
| | | | |
| 5 | Payment or honoraria for lectures, presentations, speakers bureaus, manuscript writing or educational events | X None | |
| | | | |
| | | | |
| 6 | Payment for expert testimony | X None | |
| | | | |
| | | | |
| 7 | Support for attending meetings and/or travel | X None | |
| | | | |
| | | | |
| 8 | Patents planned, issued or pending | X None | |
| | | | |
| | | | |
| 9 | Participation on a Data Safety Monitoring Board or Advisory Board | X None | |
| | | | |
| | | | |
| 10 | Leadership or fiduciary role in other board, society, committee or advocacy group, paid or unpaid | X None | |
| | | | |
| | | | |
| 11 | Stock or stock options | X None | |
| | | | |
| | | | |
| 12 | Receipt of equipment, materials, drugs, medical writing, gifts or other services | X None | |
| | | | |
| | | | |
| 13 | Other financial or non-financial interests | TICEBA GmbH | Employee, CSO |
| | | RHEACELL GmbH & Co.KG | CSO |
| | | | |

**Please place an "X" next to the following statement to indicate your agreement:**

**X I certify that I have answered every question and have not altered the wording of any of the questions on this form.**

## TREND Statement Checklist

| Paper Section/ Topic | Item No | Descriptor | Reported? ✓ | Pg # |
|---|---|---|---|---|
| **Title and Abstract** | | | | |
| Title and Abstract | 1 | • Information on how unit were allocated to interventions | ✓ | 4 |
| | | • Structured abstract recommended | ✓ | 4 |
| | | • Information on target population or study sample | ✓ | 4 |
| **Introduction** | | | | |
| Background | 2 | • Scientific background and explanation of rationale | ✓ | 6-8 |
| | | • Theories used in designing behavioral interventions | ✓ | 6-8 |
| **Methods** | | | | |
| Participants | 3 | • Eligibility criteria for participants, including criteria at different levels in recruitment/sampling plan (e.g., cities, clinics, subjects) | ✓ | 22 |
| | | • Method of recruitment (e.g., referral, self-selection), including the sampling method if a systematic sampling plan was implemented | n.a. | n.a. |
| | | • Recruitment setting | ✓ | 22 |
| | | • Settings and locations where the data were collected | ✓ | 22 |
| Interventions | 4 | • Details of the interventions intended for each study condition and how and when they were actually administered, specifically including: | ✓ | 22-23 |
| | | ○ Content: what was given? | ✓ | 22-23 |
| | | ○ Delivery method: how was the content given? | n.a. | n.a. |
| | | ○ Unit of delivery: how were the subjects grouped during delivery? | n.a. | n.a. |
| | | ○ Deliverer: who delivered the intervention? | ✓ | 22 |
| | | ○ Setting: where was the intervention delivered? | ✓ | 22 |
| | | ○ Exposure quantity and duration: how many sessions or episodes or events were intended to be delivered? How long were they intended to last? | ✓ | 23 |
| | | ○ Time span: how long was it intended to take to deliver the intervention to each unit? | ✓ | 23 |
| | | ○ Activities to increase compliance or adherence (e.g., incentives) | n.a. | n.a. |
| Objectives | 5 | • Specific objectives and hypotheses | ✓ | 8 |
| Outcomes | 6 | • Clearly defined primary and secondary outcome measures | ✓ | 23-24 |
| | | • Methods used to collect data and any methods used to enhance the quality of measurements | n.a. | n.a. |
| | | • Information on validated instruments such as psychometric and biometric properties | ✓ | 23 |
| Sample Size | 7 | • How sample size was determined and, when applicable, explanation of any interim analyses and stopping rules | n.a. | n.a. |
| Assignment Method | 8 | • Unit of assignment (the unit being assigned to study condition, e.g., individual, group, community) | ✓ | 22 |
| | | • Method used to assign units to study conditions, including details of any restriction (e.g., blocking, stratification, minimization) | n.a. | n.a. |
| | | • Inclusion of aspects employed to help minimize potential bias induced due to non-randomization (e.g., matching) | n.a. | n.a. |

## TREND Statement Checklist

| | | | | |
|---|---|---|---|---|
| Blinding (masking) | 9 | • Whether or not participants, those administering the interventions, and those assessing the outcomes were blinded to study condition assignment; if so, statement regarding how the blinding was accomplished and how it was assessed. | ✓ | 22 |
| Unit of Analysis | 10 | • Description of the smallest unit that is being analyzed to assess intervention effects (e.g., individual, group, or community) | ✓ | 24 |
| | | • If the unit of analysis differs from the unit of assignment, the analytical method used to account for this (e.g., adjusting the standard error estimates by the design effect or using multilevel analysis) | n.a. | n.a. |
| Statistical Methods | 11 | • Statistical methods used to compare study groups for primary methods outcome(s), including complex methods of correlated data | ✓ | 24 |
| | | • Statistical methods used for additional analyses, such as a subgroup analyses and adjusted analysis | ✓ | 24 |
| | | • Methods for imputing missing data, if used | ✓ | 23 |
| | | • Statistical software or programs used | ✓ | 24 |
| **Results** | | | | |
| Participant flow | 12 | • Flow of participants through each stage of the study: enrollment, assignment, allocation, and intervention exposure, follow-up, analysis (a diagram is strongly recommended) | ✓ | Fig. 1B |
| | | ○ Enrollment: the numbers of participants screened for eligibility, found to be eligible or not eligible, declined to be enrolled, and enrolled in the study | ✓ | Fig. 1B |
| | | ○ Assignment: the numbers of participants assigned to a study condition | ✓ | Fig. 1B |
| | | ○ Allocation and intervention exposure: the number of participants assigned to each study condition and the number of participants who received each intervention | ✓ | Fig. 1B |
| | | ○ Follow-up: the number of participants who completed the follow-up or did not complete the follow-up (i.e., lost to follow-up), by study condition | ✓ | Fig. 1B |
| | | ○ Analysis: the number of participants included in or excluded from the main analysis, by study condition | ✓ | Fig. 1B |
| | | • Description of protocol deviations from study as planned, along with reasons | ✓ | 9 |
| Recruitment | 13 | • Dates defining the periods of recruitment and follow-up | ✓ | 9 |
| Baseline Data | 14 | • Baseline demographic and clinical characteristics of participants in each study condition | ✓ | Table 1 |
| | | • Baseline characteristics for each study condition relevant to specific disease prevention research | n.a. | n.a. |
| | | • Baseline comparisons of those lost to follow-up and those retained, overall and by study condition | n.a. | n.a. |
| | | • Comparison between study population at baseline and target population of interest | n.a. | n.a. |
| Baseline equivalence | 15 | • Data on study group equivalence at baseline and statistical methods used to control for baseline differences | n.a. | n.a. |

## TREND Statement Checklist

| | | | | |
|---|---|---|---|---|
| Numbers analyzed | 16 | • Number of participants (denominator) included in each analysis for each study condition, particularly when the denominators change for different outcomes; statement of the results in absolute numbers when feasible | ✓ | Figures and Tables |
| | | • Indication of whether the analysis strategy was “intention to treat” or, if not, description of how non-compliers were treated in the analyses | ✓ | 24 |
| Outcomes and estimation | 17 | • For each primary and secondary outcome, a summary of results for each estimation study condition, and the estimated effect size and a confidence interval to indicate the precision | ✓ | 9-13 |
| | | • Inclusion of null and negative findings | ✓ | 9-13 |
| | | • Inclusion of results from testing pre-specified causal pathways through which the intervention was intended to operate, if any | n.a. | n.a. |
| Ancillary analyses | 18 | • Summary of other analyses performed, including subgroup or restricted analyses, indicating which are pre-specified or exploratory | n.a. | n.a. |
| Adverse events | 19 | • Summary of all important adverse events or unintended effects in each study condition (including summary measures, effect size estimates, and confidence intervals) | ✓ | 12-13, Table 2 |
| **DISCUSSION** | | | | |
| Interpretation | 20 | • Interpretation of the results, taking into account study hypotheses, sources of potential bias, imprecision of measures, multiplicative analyses, and other limitations or weaknesses of the study | ✓ | 14-21 |
| | | • Discussion of results taking into account the mechanism by which the intervention was intended to work (causal pathways) or alternative mechanisms or explanations | ✓ | 14-21 |
| | | • Discussion of the success of and barriers to implementing the intervention, fidelity of implementation | ✓ | 14-21 |
| | | • Discussion of research, programmatic, or policy implications | ✓ | 20-21 |
| Generalizability | 21 | • Generalizability (external validity) of the trial findings, taking into account the study population, the characteristics of the intervention, length of follow-up, incentives, compliance rates, specific sites/settings involved in the study, and other contextual issues | ✓ | 20-21 |
| Overall Evidence | 22 | • General interpretation of the results in the context of current evidence and current theory | ✓ | 14-21 |

*From:* Des Jarlais, D. C., Lyles, C., Crepaz, N., & the Trend Group (2004). Improving the reporting quality of nonrandomized evaluations of behavioral and public health interventions: The TREND statement. *American Journal of Public Health*, 94, 361-366. For more information, visit: http://www.cdc.gov/trendstatement/
